# The dynamic impact of investor climate sentiment on the crude oil futures market: Evidence from the Chinese market

**DOI:** 10.1371/journal.pone.0314579

**Published:** 2025-02-12

**Authors:** Wenwen Liu, Miaomiao Tang, Peng Zhao

**Affiliations:** School of Economics, Xihua University, Chengdu, Sichuan, China; Bucharest University of Economic Studies: Academia de Studii Economice din Bucuresti, ROMANIA

## Abstract

Climate risk has become a hot topic of global concern. This paper aims to explore the impact of investor climate sentiment (ICS) on China’ s crude oil futures market, covering the period from March 27, 2018, to December 30, 2022. Firstly, this paper employs the Thermal Optimal Path (TOP) method and discovers that the guiding effect of ICS on the volatility of crude oil futures (RV^oil^) intensifies over time, progressively becoming a pivotal factor in determining volatility. Secondly, based on the lead-lag relationship between ICS and RV^oil^, this study divides the sample period into five stages and confirms through the HAR model that ICS has a significant inhibitory effect on crude oil volatility during the guiding phase. In addition, incorporating ICS into the HAR model not only improves the model’ s goodness of fit but also significantly reduces the prediction error in out-of-sample forecasts. Finally, by comparing with the full-sample analysis, the volatility prediction results of the segmented samples show that during the guiding phase, the predictive power of ICS for crude oil market volatility is significantly improved. Even in the non-guiding phase, ICS can reduce the prediction error to a certain extent. This result further highlights the advantages of the TOP method in revealing the impact of ICS on the prediction of crude oil volatility.

## Introduction

The global climate crisis has been a pressing issue for decades, with its effects permeating all aspects of human life and economic activities. Energy commodities play an indispensable role in the growth of the real economy [1, 2]. Climate change is bound to have a significant impact on the volatility of energy market prices [3, 4]. Extreme weather events and natural disasters caused by climate change pose serious risk challenges to energy-intensive companies [5].

There are several convincing reasons to study the dynamic relationships between climate risk and crude oil prices volatility. First, it is often of interest to identify the key factors affecting the fluctuation of oil prices and predict volatility. As an important energy commodity, studying the impact of climate risk on crude oil price fluctuations is highly relevant. Climate risk affects the volatility of crude oil prices by influencing economic activities, altering the energy structure, and impacting financial markets [6–9].

Second, Previous studies tend to focus on WTI and Brent [10–12]. The oil price shock has a significant impact on the economic activities of Asian countries in the short term, and has a more positive macroeconomic impact on some Asian countries [13, 14]. China is one of the world’s largest consumers and importers of crude oil, fluctuations in China’s crude oil market can have a significant impact not only on the Asian market but also on the global market. China’s market is particularly sensitive to shifts in climate risk, because China is quite seriously affected by climate disasters. Therefore, studying the impact of climate change on the fluctuations in China’s crude oil market is crucial for policymakers, investors, and energy companies.

Third, this paper employs the nonlinear TOP model to delve into the dynamic leading relationship between climate sentiment and fluctuations in crude oil prices [15]. The current literature predominantly employs traditional econometric models, including linear regression [16], nonlinear regression [17], SVAR models [18], and Granger causality tests [19] etc. These methods mainly rely on linear or nonlinear parameter estimation. The TOP model is a non-parametric estimation technique used to reveal the dynamic lead-lag relationship between complex sequences. One of the main advantages of the TOP methods is that they are able to identify the time-varying lead-lag structure between two time series. This feature means that one does not need to differentiate the non-stationary series, thus preserve all the information of the original time series [20, 21].

Finally, predicting the volatility of the crude oil prices is a key means to gain insight into volatility trends and manage financial risks [10, 22–26]. Based on the dynamic results of the TOP model, we incorporate climate risk into the Heterogeneous Autoregressive model (HAR) for in-sample and out-of-sample volatility predictions. HAR model is built on high-frequency data, which is informative and can provide more rapid decision support for market traders and investors. Numerous empirical studies have shown that the HAR volatility model and its extended model outperform GARCH-type models and the SV model in terms of performance [27–31].

Based on the aforementioned background, the purpose of this study is to quantify some of the climate risks faced by China by constructing the Investor Climate Sentiment (ICS) index. It analyzes how climate change affects the volatility of China’ s crude oil market from an investor’s perspective, and further provides predictive analysis of future trends in the crude oil market. According to behavioral finance theory, investor sentiment can lead to additional volatility in financial markets and influence asset pricing [32–38]. Investor sentiment can predict the future direction of crude oil prices and volatility [39–42]. Scholars have conducted empirical research and found that online information can influence investor sentiment, which in turn affects investment decisions [43–46]. Moreover, when climate information is taken into account, it has a significant impact on energy price volatility in inefficient markets [47].

We employ the TOP method to uncover the lead-lag relationship between Investor Climate Sentiment (ICS) and the volatility of crude oil futures, and demonstrate the dynamic evolution of this relationship over time. In the early stages of the pandemic, the influence of crude oil futures volatility on ICS increased, but as time progressed, ICS gradually became the key factor guiding the volatility of crude oil futures, with its leading order progressively strengthening. This phenomenon indicates that investors’ attention to the issues of the pandemic and climate change has undergone significant changes, reflecting the market investors ‘ongoing focus and emphasis on the long-term impacts of climate change.

This study, based on the lead-lag relationship between Investor Climate Sentiment (ICS) and crude oil futures volatility (RV), subdivides the sampling period into five stages: the first stage where RV and ICS alternately lead (from March 27, 2018, to November 29, 2019), the second stage where RV leads ICS (from December 2, 2019, to February 5, 2020), the third stage where ICS leads RV (from February 6, 2020, to June 22, 2020), the fourth stage where RV leads ICS again (from June 23, 2020, to September 7, 2021), and the fifth stage where ICS reasserts its leadership over RV (from September 8, 2021, to December 30, 2022). This subdivision allows for a more accurate analysis of the impact of ICS on the volatility of the crude oil market during different phases.

Incorporating investor climate sentiment (ICS) into the in-sample estimation of the HAR model significantly enhances the model’ s goodness of fit. In the out-of-sample prediction of crude oil volatility, the integration of ICS significantly reduces the prediction error, indicating that ICS, as an important variable for predicting crude oil futures volatility, provides additional predictive information.

The marginal contributions of this paper are mainly reflected in the following aspects: First, this study uses text analysis method to successfully construct a more accurate and detailed investor climate sentiment index based on the collected climate-related information. It fills the gap in the research on investor climate sentiment in China and provides a new perspective and method for the follow-up research. Secondly, compared to traditional econometric models, we employ a non-parametric statistical physics model to delve into the dynamic relationship between ICS and the volatility of INE crude oil futures. Third, guided by the TOP method, this study investigates the impact of incorporating the ICS on the predictive performance of the HAR model and its extensions through the segmentation of sample data. The research findings indicate that the incorporation of the ICS significantly optimizes the model’s performance and effectively reduces the model’s prediction errors.

The structure of this paper is as follows: Section 1 is the introduction. Section 2 introduces the relevant literature. In Section 3, This study elaborates on the construction process of the ICS, the TOP model, and the HAR-RV model. Section 4 describes the data sources and data description. Section 5 conducts the empirical analysis of the model. The final section presents relevant policy recommendations based on the empirical results.

## Literature review

### The impact of climate change on financial markets

In recent years, the impact of climate change on financial stability has received increasing scholarly attention. Most scholars believe that the emission of greenhouse gases will lead to the frequent occurrence of natural disasters, and if the government releases appropriate policies to mitigate climate change, it will adversely affect the operation of the macroeconomy. As a result of the macroeconomic recession, it will further increase the uncertainty of the financial system operation and impact the stability of the financial markets [48–51].

The risks posed by climate change can be categorized into two types, one being physical risks and the other being transformational risks. Physical risks can be thought of primarily as the impact of natural disasters brought about by climate change on the financial system by causing damage to the economy as a whole [52–57]. Regarding the transformational risks of climate change, the academics argue that the implementation of climate change policies will strand assets in carbon-intensive industries, especially in the energy sector. The loss of stranded assets to these industries is incalculable, which will not only lead to economic losses, credit defaults, and a decline in the market value of enterprises, but will even trigger a chain effect leading to a financial crisis [58, 59].

Since China signed the Paris Agreement in 2016, the issue of climate change has received extensive attention from domestic scholars. Related studies have pointed out that the physical risks of climate change through extreme weather events can have a negative impact on China’s economic development and financial stability [60]. With regard to the impact of climate policy, some scholars believe that green financial policies have a negative effect on total socio-economic output, and that financial policies can improve the capital adequacy ratio of the financial sector in the short term, while carbon tax policies are beneficial to the capital adequacy ratio of banks only in the longer term [61]. The implementation of green finance helps to mitigate the shock to bank risk, and the shock to financial risk from a carbon abatement path scenario based on the allocation of carbon abatement responsibility is smaller than that from a carbon abatement path scenario with the same proportion [62]. In particular, [63] used SVAR model to analyze that climate risk does have a positive impact on banking risk and is heterogeneous among different types of banks, and the sensitivity of different banks to climate risk shocks is not the same. At present, there are few domestic studies related to the impact of climate risk on China’s financial market, and most of the empirical studies mainly focus on the impact of climate policies on macroeconomies [64–67]. In contrast, the research in this paper is more oriented to the micro level, and mainly analyzes the change of investors’ (individual) behavioral perspectives as a result of climate change, which in turn affects the volatility of individual markets.

### Forecasting volatility using realized volatility

A substantial body of literature has demonstrated the significant efficacy of the GARCH model in predicting volatility in the crude oil market [68–72]. However, due to challenges associated with accessing high-frequency financial data during its early stages, scholars were constrained to focus solely on low-frequency volatility prediction using GARCH-type models. However, with the increasing maturity of information technology, the acquisition of high-frequency financial data has become more convenient. [73] was the first to propose using realized volatility as a measure of stock market volatility, focusing on the foreign exchange market as their research subject. They discovered that when sampling frequency was high, the sum of squared returns of financial assets could be considered an unbiased estimate of true volatility over a given time period. [74] introduced the Heterogeneous Autoregressive Realized Volatility (HAR-RV) model based on heterogeneous market theory. This model utilizes realized volatility at three different frequencies—daily, weekly, and monthly—to describe heterogeneity in short-term, medium-term, and long-term traders’ trading behavior. Despite its apparent simplicity, this model effectively captures the long memory characteristics observed in price volatility of financial assets. [75] utilized the double power variation theory to decompose realized volatility into integral variance and jump variance, thereby developing the HAR-RV-CJ model that significantly enhances accuracy in volatility prediction. [76] extended this approach by introducing the LHAR-CJ model to simultaneously characterize both jump effect and leverage effect’s influence on volatility within HAR-RV-VJ framework.

Based on the findings from the aforementioned literature, it can be inferred that the HAR model outperforms the GARCH model in terms of volatility prediction. Furthermore, previous literature on predicting crude oil market volatility often neglected the impact of external factors and solely relied on crude oil market data. However, considering the significant role of investor sentiment in various events and its reflection in crude oil market volatility, it is scientifically justifiable to incorporate investor sentiment. Based on this, we incorporated the ICS index into the HAR-RV model proposed by Corsi [74] to predict the volatility of China’ s crude oil market.

## Methodology

### TOP (Thermal Optimal Path) modeling approach

The thermal optimal path (TOP) method is originally developed by Sornette [15] and Zhou [77]. They used it to explore the lead-lag relationship between different financial assets. They studied the monthly returns linkages among the S&P 500 Index and the U.S. federal government funds rate and the monthly changes in 10 different national bond yields. By introducing the distance matrix, the classical economic problem is transformed into a probability transmission model and eventually dynamic relationship can be obtained through the partition function. The exact details of the implementation of the method are presented below.

Firstly, assume there are two normalized time series x(t_1_): t_1_ = 1,2,…,T_1_ and y(t_2_): t_1_ = 1,2,…,T_2_. For convenience, we let T_0_ be the length of the two time series. The d(t_1_, t_2_) indicates distance between the two time series: x(t_1_) and y(t_2_).


$$d(t_{1},t_{2})=|x(t_{1})-y(t_{2})|$$ (1)

To discover a mapping relation between t_1_ and t_2_, the TOP method is used, that is, t_2_ = ϕ(t_1_), which can minimize the distance matrix.


$$\phi^{*}(t_{1})=\underset{\phi (t_{1})}{\arg\;min}E=\underset{\phi (t_{1})}{\arg\;min}\sum_{t_{1}=0}^{T_{0}}\left|x(t_{1})-y(\phi (t_{1}))\right|$$ (2)

Where E is the total distance along path φ(t_1_) between two time series. The mapping relation is subject to an additional restriction in order to guarantee the path’s continuity: $0\leq \phi (t_{1}+1)-\phi (t_{1})\leq 1$.

Since most of the data in the real study are non-stationary and contain a large amount of white noise, for the convenience of the study [20, 78–81], We first convert the coordinates (t_1_, t_2_) into (t, l).


$$\left\{ \begin{array}{c} t=t_{1}+t_{2}\\ l=t_{2}-t_{1} \end{array}\right.$$ (3)

Where t is perpendicular to l and lies in the major diagonal of the (t_1_, t_2_) coordinate system. The dynamic lead-lag relationship between two series is quantified by l(t), and a positive (negative) l(t) indicates that the first time series, x(t_1_), leads (lags) the second time series, y(t_2_). Consequently, we can determine the ideal thermal path $\hat{l}(t)$ by:

$$\hat{l}(t)=\sum l\cdot \frac{w(t,l)}{w(t)}$$ (4)

In which the partition function Boltzmann factors over all routes leading to (t, l) are added to form w(t, l), and w(t) = ∑_l_w(t, l). Consequently, the chance that a path will be at position l at time t can be expressed as w(t, l)/w(t). A positive $\hat{l}(t)$ indicates that the first time series, x(t_1_), leads the second, y(t_2_), and vice versa.

A plausible path that leads to (t, l) can originate from (t−1, l−1) vertically, (t−1, l +1) horizontally, or (t−2, l) diagonally in order to maintain the direction of time required by causality. Consequently, w(t, l) can be calculated in a recursive manner as shown below:

$$w(t,l)=(w(t-1,l-1)+w(t-1,l+1)+w(t-2,l))e^{-d(t,l)/Tem}$$ (5)

Where Tem is a temperature parameter that manages the degree of noise effect.

### Construction of volatility models

Volatility prediction has always been a central issue in financial theory and practical research. A significant body of research has shown that the HAR model outperforms the Stochastic Volatility (SV) model and the GARCH model in out-of-sample prediction [29–31]. Additionally, the research by Andersen and others further strengthens this viewpoint [27, 28]. Their study verifies that models based on realized volatility are significantly more accurate than GARCH models in predicting volatility.

In light of the above, this section will provide a detailed introduction to the HAR-RV model proposed by Corsi [74], as well as its extension, the HAR-RV-ICS model.

Andersen and Bollerslev [82] define the realized volatility (RV) on day t as the sum of the squares of all high-frequency returns on that day. The formula is as follows:

$${RV}_{t}=\sum_{i=1}^{n_{t}}r_{t,i}^{2}$$ (6)

Where $r_{t,i}=100*ln(P_{t,i}/P_{t,i-1})$ represents the i-th high-frequency return on day t; P is the futures closing price; n_t_ indicates the total number of high-frequency returns on day t.

Referencing Corsi’s study [74], the HAR-RV model under the framework of high-frequency volatility can be formulated as follows:

$$\bar{{RV}_{t+s}}=\omega_{0}+\omega_{1}{RV}_{t-1}+\omega_{2}{RV}_{t-5}^{w}+\omega_{3}{RV}_{t-22}^{m}+\mu_{t+s}$$ (7)

Where $\bar{{RV}_{t+s}}=({RV}_{t+1}+{RV}_{t+2}+\cdots +{RV}_{t+s})/s,s=(1,5,22)$ represents the 1-day, 1-week and 1-month future RV, respectively; Where ${RV}_{t-i}=({RV}_{t}+{RV}_{t-1}+\cdots +{RV}_{t-(i-1)})/i,i=(5,22)$ denotes the weekly and monthly RV.

The research by Andersen, et al. [75] reveals that the logarithmic HAR model surpasses the traditional HAR model in terms of fitting capability. Furthermore, transforming the realized volatility (RV) into its logarithmic form significantly enhances the accuracy of predicts [83–85]. Based on this, this paper employs the logarithmic HAR model to predict the volatility of the crude oil market. The corresponding HAR-RV model is specified as follows:

$$log(\bar{{RV}_{t+s}})=\omega_{0}+\omega_{1}log({RV}_{t-1})+\omega_{2}log({RV}_{t-5}^{w})+\omega_{3}log({RV}_{t-22}^{m})+\mu_{t+s}$$ (8)

In the final part of this section, following the research of Liu, et al. [38], we have incorporated the Investor Climate Sentiment Index (ICS) into the logarithmic HAR model with the aim of investigating whether ICS carries any additional predictive value. Similar to Eq (7), this study further takes into account the monthly and weekly averages of these variables. Based on this, the extended model, which includes the ICS index—HAR-RV-ICS—is presented as follows:

$$log(\bar{{RV}_{t+s}})=\omega_{0}+\omega_{1}log({RV}_{t-1})+\omega_{2}log({RV}_{t-1}^{w})+\omega_{3}log({RV}_{t-1}^{m})+\gamma_{1}{ICS}_{t-1}+\gamma_{2}{ICS}_{t-5}^{w}+\gamma_{3}{ICS}_{t-22}^{m}+\mu_{t+s}$$ (9)

Where ${ICS}_{t-1},\;{ICS}_{t-5}^{w}$, and ${ICS}_{t-22}^{m}$ represent the Investor Climate Sentiment Index lagged by 1 day, 1 week, and 1 month, respectively.

## Data description

### Investor climate sentiment

According to previous studies, investor sentiment is mainly reflected in the views of retail investors. Retail investors are more susceptible to behavioral bias than institutional investors [36]. Therefore, when constructing the investor sentiment climate, it is necessary to choose professional large financial review forums as our data sources. As one of the most influential social media in China, The East Money Stock Bar Forum has the largest stock bar user base and the highest page views. There have been many studies using the Easy Money Stock Bar Forum data sources [86–89]. Based on this, in this section, we mainly select the comments related to climate change on the Eastern Money Stock Bar Forum from January 2014 to December 2022 as the analysis object. And the investor climate sentiment index is constructed through the following four steps: Firstly, use Python web scraping technology to collect stock bar review information. Secondly, the obtained review information is preprocessed. Thirdly, a machine learning model is used to analyze the sentiment of the pre-processed comments. Finally, the investor climate sentiment index is constructed.

This research utilizes Python’ s requests and Beautiful Soup libraries to extract 4.8 million records from the East Money Stock Bar forum, with a focus on post titles for sentiment analysis, as they directly and effectively express investors’ viewpoints.

To create a reliable investor sentiment indicator from the collected posts, data processing is essential. It involves removing repetitive and ad-laden content, using the Jieba library for word segmentation with a stop word list, and manually categorizing a random sample of 10,000 posts from 4 million into positive and negative sentiment groups. The model evaluates mood change, with values closer to 1 indicating positive sentiment and those near -1 indicating negative sentiment due to events.

Due to the independence of feature words, the Naive Bayes method is an ideal choice for sentiment analysis because of its simplicity and efficiency [90–92]. Accordingly, when initially constructing the investor climate sentiment index, we selected the Naive Bayes classification technique. By utilizing the Python SnowNLP library, we conducted sentiment training on 10,000 text vectors labeled with sentimental tags. The model evaluation results show an accuracy rate of 75%, indicating its good classification performance. Generally, if the accuracy rate of a classification model can reach 70%, it can be considered to have achieved an effective classification result.

Following the application of the Naive Bayes algorithm to score the text information of the entire sample set, this paper conducted a statistical analysis of the sentiment categories for each sample and compiled the daily investor sentiment scores and investor climate sentiment scores. To ensure that climate sentiment reflects a broader range of investor emotions, it was orthogonalized with the overall sentiment of the East Money stock bar forum, following the method of Santi [93]. The following equation was estimated using ordinary least squares (OLS) regression:

$${CSent}_{t}=\alpha +\beta {Sent}_{t}+\varepsilon$$ (10)

Where CSent_t_ is the daily climate sentiment score; Sent_t_ is the daily average sentiment score of all posts from the East Money stock bar. The orthogonal Investor Climate Sentiment (ICS) is calculated as follows:

$${ICS}_{t}={CSent}_{t}-\hat{\beta }{Sent}_{t}$$ (11)

**Fig 1:**
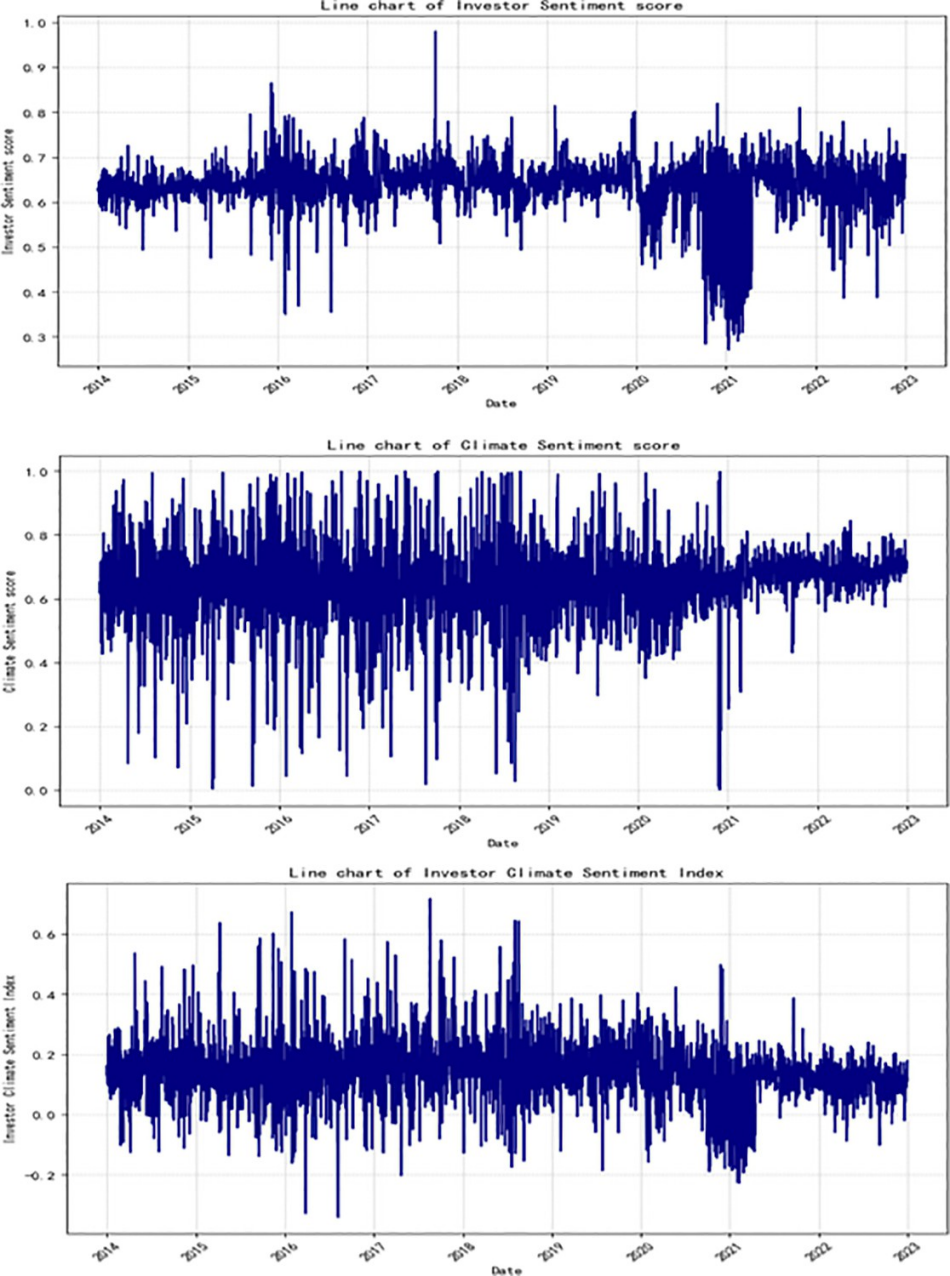
Line chart of sentiment score and investor climate sentiment index. (a) Investor Sentiment Score. (b) Investor Climate Sentiment Score. (c) Investor Climate Sentiment Index.

**Fig 1A**–**1C** respectively display the trends in investor sentiment scores, climate sentiment scores, and the investor climate sentiment index. Observing the charts, it is evident that the changes in these three sets of data exhibit a clear characteristic of clustering. Over most periods, the volatility of these three sets of data demonstrate a high degree of consistency. Significantly, since the Intergovernmental Panel on Climate Change (IPCC) published its Fifth Assessment Report in 2013, investor climate sentiment has been subject to market volatility s and unpredictability. After the release of the report, investor sentiment towards climate change showed a downward trend, particularly from the end of 2014 to 2016, during which the decline was more pronounced.

### Crude oil futures

This study selects China’s crude oil futures price data from March 27, 2018, to December 30, 2022, as the research object for empirical analysis. The data includes INE crude oil daily futures prices and INE crude oil 5-minute high-frequency futures prices, covering 1,156 trading days with a total of 124,665 observations, all sourced from the Wind database. For the selection of crude oil futures data, this article uses the data of the main contract. The main contract represents the peak of trading volume and open interest on the market for the day, and it is actively traded with strong maneuverability. Furthermore, in view of the non-uniformity of high-frequency futures data in terms of time distribution, this study draws on the research methods of scholars such as Liu, et al. [38] and excludes high-frequency data samples before 9:30 AM and after 3:00 PM. This treatment is aimed at ensuring the accuracy and reliability of the constructed realized volatility (RV).

**Fig 2:**
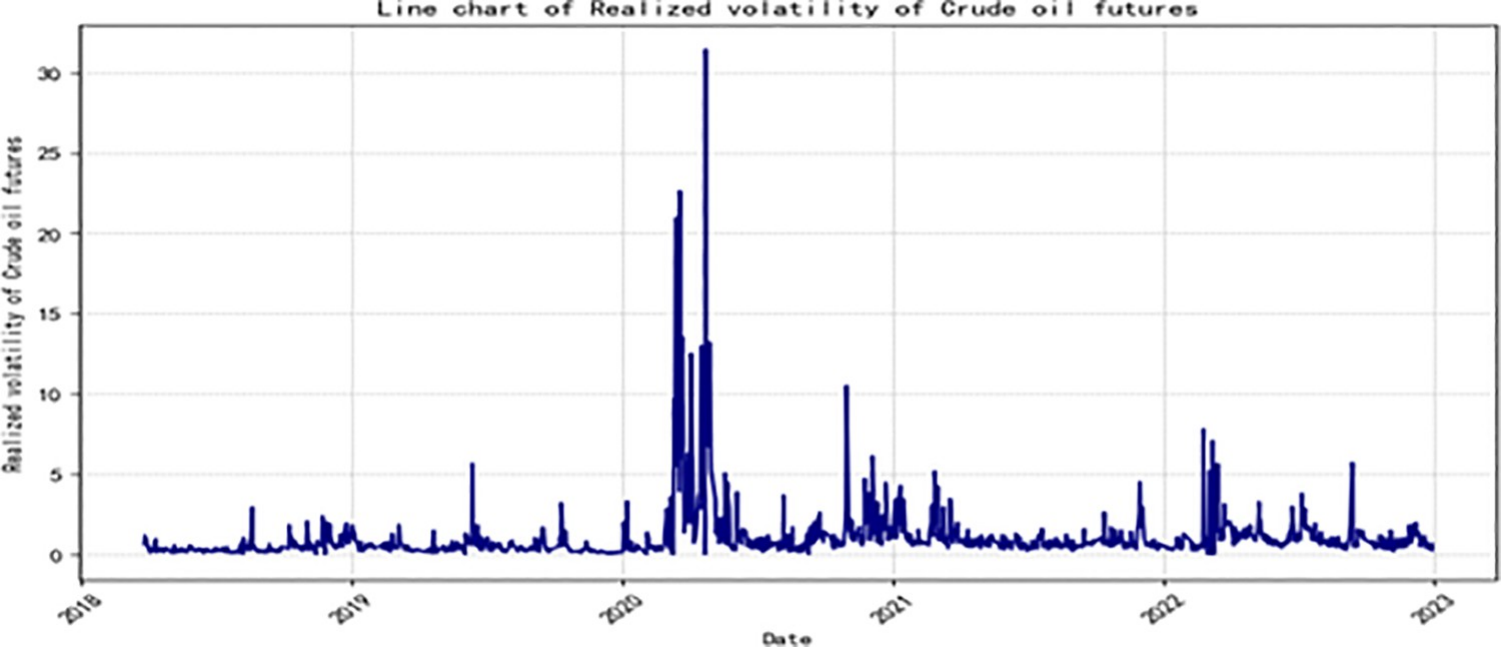
Line chart of Realized Volatility (RV) of crude oil futures.

**Fig 2** shows the trend of realized volatility (${RV}_{t}=\sum_{i=1}^{n_{t}}r_{t,i}^{2}$, Eq (6)) for crude oil futures. It can be observed that the volatility exhibits a dynamic pattern of first decreasing and then increasing. At the same time, the data also displays a significant clustering phenomenon. Additionally, it is worth noting that during the full outbreak of the COVID-19 pandemic in 2020, the sharp decline in market prices resulted in a significant increase in volatility.

### Descriptive statistics of variables and stationarity test

This paper synchronizes the daily data of the Investor Climate Sentiment Index with the daily data of the realized volatility of crude oil futures, and uniformly adopts the data sample from March 27, 2018, to December 30, 2022. The detailed statistical results after synchronization are presented in **Table 1**. In terms of the realized volatility of crude oil futures, the skewness of ${RV}^{oil},\;{RV}_{t-1}^{oil},\;{RV}_{w}^{oil},\;{RV}_{m}^{oil}$ all exceed 0.5, with kurtosis far surpassing 2, displaying statistical characteristics of a sharp peak, thick tails, and a rightward skew. On the other hand, the Investor Climate Sentiment Index (ICS) and its lags ICS_t−1_, weekly ICS_w_, and monthly ICS_m_ all have skewness values less than -0.5, with kurtosis also far exceeding 2, indicating a significant sharp peak and thick tail characteristic, and the data distribution is skewed to the left. All data do not conform to the characteristics of a normal distribution.

**Table 1:** Descriptive statistics of variables and stationarity test.

Variable	Mean	Sd	Skewness	Kurtosis	Min	Max	ADF Value
**RV** ^**oil**^	0.966	1.349	4.100	23.072	0.007	9.545	-20.884
${RV}_{t-1}^{oil}$	0.966	1.350	4.098	23.051	0.007	9.545	-17.563
${RV}_{w}^{oil}$	0.967	1.070	3.611	19.422	0.068	8.945	-4.590
${RV}_{m}^{oil}$	0.972	0.902	2.885	12.536	0.100	5.450	-3.456
**ICS**	1156	0.111	0.0954	-0.540	4.725	-0.227	-17.282
**ICS**_**t−1**_	0.111	0.093	-0.709	4.149	-0.171	0.341	-16.902
**ICS**_**w**_	0.111	0.075	-1.392	5.553	-0.165	0.323	-4.130
**ICS**_**m**_	0.111	0.066	-1.633	5.723	-0.125	0.250	-3.578

Note: Z(t) = ADF Value, Z(t) < -3.430 indicates that the test value is stationary at the 1% significance level; -3.430 < Z(t) < -2.860 indicates that the test value is stationary at the 5% significance level; -2.860 < Z(t) < -2.570 indicates that the test value is stationary at the 10% significance level; ${RV}^{oil},\;{RV}_{t-1}^{oil},\;{RV}_{w}^{oil}$, and ${RV}_{m}^{oil}$ represent the realized volatility of crude oil futures for the current period, one-period lag, one-week lag, and one-month lag, respectively; ICS, ICS_t−1_, ICS_w_, and ICS_m_ represent the Investor Climate Sentiment index for the current period, one-period lag, one-week lag, and one-month lag, respectively.

In the results of the stationarity test, the ADF values of all variables are lower than the critical value of -3.430, indicating that these variables meet the requirements of stationarity.

## Empirical analysis

### Empirical analysis of Thermal Optimal Path method (TOP)

**Fig 3:**
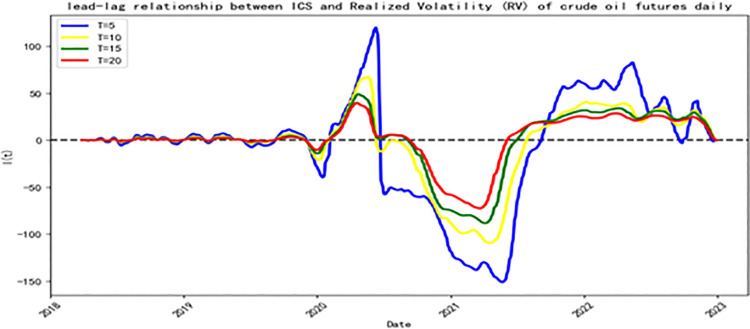
The relationship between Investor Climate Sentiment (ICS) and Realized Volatility of crude oil futures (RV). **Note:** In the chart, values above the zero line indicate that the Investor Climate Sentiment (ICS) index leads the Realized Volatility (RV) of Crude oil futures, and vice versa.

**Fig 3** illustrates the leading-lag relationship between the Investor Climate Sentiment (ICS) index and the realized volatility of crude oil futures (RV^oil^). From March 2018 to November 2019, there was a mutual leading relationship between ICS and RV^oil^, but this relationship was not significant, indicating that the impact of climate change on China’ s crude oil futures market was relatively limited. However, during the initial outbreak of the COVID-19 epidemic from December 2019 to February 2020, a significant change occurred in the leading relationship between RV^oil^ and ICS, with RV^oil^’ s dominant role over ICS significantly enhanced, reaching a lead order of 39. The COVID-19 pandemic, as a global sudden incident, quickly captured the market’ s attention, triggering significant fluctuations in market sentiment. According to the limited attention theory in behavioral finance [94, 95], this shift in attention caused investors to focus more on short-term economic impacts and market fluctuations, while relatively neglecting the long-term issues of climate change. Furthermore, the attentional focus brought about by the pandemic also shifted investors’ risk preferences, leading them to prioritize the divestment from high-risk assets, a trend that was initially manifest in the pricing of assets like crude oil [96]. Hence, during periods of intense focus, the volatility of crude oil futures (RV^oil^) guided Investor Climate Sentiment (ICS).

From February 6, 2020, to June 22, Investor Climate Sentiment (ICS) significantly led the volatility of crude oil futures (RV^oil^). This trend indicates that market participants are increasingly attaching importance to climate change issues and incorporating them into the core considerations of their investment decisions. As suggested by Prospect Theory [97, 98], when faced with the increased market uncertainty caused by the COVID-19 pandemic, investors typically re-evaluate the relationship between risk and return. This re-evaluation may lead investors to pay more attention to the long-term risks associated with climate change, which demonstrates sensitivity to Investor Climate Sentiment (ICS) in investment decisions within energy markets such as crude oil. Consequently, the volatility of crude oil futures, to some extent, reflects investors’ expectations of future climate change impacts and adjustments to their risk preferences.

During the peak of the pandemic from June 23, 2020, to September 7, 2021, the volatility of crude oil futures (RV^oil^) significantly led investor climate sentiment (ICS), a phenomenon that may have been influenced by herding behavior [99]. Under the renewed impact of the pandemic event, market participants may be more inclined to follow herd behavior, leading to a significant increase in the lead of crude oil futures volatility (RV^oil^). With the pandemic and its impact on the global economy once again becoming the focus, investors’ attention is refocused on the associated risks, intensifying the herding effect. Therefore, during this period, the dominance of crude oil futures volatility (RV^oil^) over ICS significantly increased, reflecting the market’ s immediate response to pandemic-related risks and the amplification effect of herd behavior. After September 7, 2021, Investor Climate Sentiment (ICS) once again guided the volatility of crude oil (RV^oil^), with the maximum order of guidance reaching 82.

In summary, this study employs the TOP method to uncover the lead-lag relationship between Investor Climate Sentiment (ICS) and the volatility of crude oil futures (RV^oil^), and demonstrates the dynamic evolution of this relationship over time. In the early stages of the pandemic, the influence of crude oil futures volatility (RV^oil^) on ICS increased, but as time progressed, ICS gradually became the key factor guiding the volatility of crude oil futures (RV^oil^), with its leading order progressively strengthening. This phenomenon indicates that investors’ attention to the issues of the pandemic and climate change has undergone significant changes, reflecting the market investors’ ongoing focus and emphasis on the long-term impacts of climate change.

### Empirical analysis of the HAR-RV and HAR-RV-ICS models

The prediction of financial market volatility has always been a focus of attention for financial researchers and practitioners. Based on the studies of Liu, et al. [38] and Gong, et al. [47], this study further explores whether Investor Climate Sentiment (ICS) can provide additional information for the prediction of crude oil futures volatility. To this end, we estimates the log-HAR model using Ordinary Least Squares (OLS) throughout the entire sample period and included the ICS index as a predictive variable in the model. Nevertheless, the studies by the aforementioned two scholars primarily employ full-sample analysis, which may overlook potential structural changes within the data. Therefore, the first part of this section focuses on a full-sample analysis, primarily examining whether Investor Climate Sentiment (ICS) contains additional information for volatility prediction. In the second part, this paper will explore whether Investor Climate Sentiment (ICS) can affect the volatility of the crude oil futures market at different leading stages. Moreover, this study follows the approach of Liu, et al. [38], using the indicators ICS_t−1_, ICS_w_ and ICS_m_ to assess the impact of Investor Climate Sentiment (ICS) on the short-term, medium-term, and long-term volatility of the crude oil futures market, respectively.

### HAR model full-sample analysis

**Table 2** summarizes the parameter estimation results of the HAR-RV model for the prediction of oil market volatility under different predict horizons for the full sample. The estimation results of the volatility model show that the coefficients of the weekly ICS index and monthly ICS index (i.e., ICS_w_ and ICS_m_) are significantly negative at the 10% and 5% significance levels. This indicates that the weekly OA index and monthly OA index contain in-sample predictive information about the weekly and monthly volatility of the oil market. Simultaneously, the negative regression coefficients also suggest that investor climate sentiment (ICS) may have a dampening effect on the medium-term and long-term volatility of the crude oil market. Additionally, the HAR-RV-ICS model, which incorporates investor climate sentiment (ICS), has a higher R-squared value than the HAR-RV model. This indicates that the HAR-RV-ICS model can explain and capture the RV dynamics in the oil market more accurately than the HAR-RV model.

**Table 2:** Full-sample estimation of the HAR model.

Subsample	Variable	Panel A		Panel B		Panel C	
		I	II	I	II	I	II
**2018.3.27–2022.12.30 (Full Sample)**	${RV}_{t-1}^{oil}$	0.151***	0.144***	0.158***	0.152***	0.092**	0.085**
		(3.43)	(3.27)	(4.40)	(4.24)	(2.37)	(2.22)
	${RV}_{w}^{oil}$	0.501***	0.497***	0.444***	0.441***	0.343***	0.339***
		(7.56)	(7.52)	(8.23)	(8.21)	(5.87)	(5.89)
	${RV}_{m}^{oil}$	0.161***	0.140**	0.240***	0.216***	0.281***	0.239***
		(2.96)	(2.54)	(5.40)	(4.80)	(5.85)	(4.96)
	**ICS**_**t−1**_		0.017		0.154		0.026
			(0.09)		(1.05)		(0.16)
	**ICS**_**w**_		0.264		0.034		-0.527*
			(0.83)		(0.13)		(-1.90)
	**ICS**_**m**_		-0.661**		-0.605**		-0.187
			(-2.17)		(-2.45)		(-0.70)
	**C**	0.074***	0.137***	0.093***	0.162***	0.191***	0.302***
		(3.53)	(3.98)	(5.44)	(5.78)	(10.31)	(10.07)
	**R** ^**2**^	0.519	0.523	0.630	0.636	0.508	0.525

**Note:** T-statistics in parentheses; The asterisks

*, ** and *** denote 10%, 5% and 1% levels of significance, respectively; I and II denote the in-sample estimation results of the logarithmic HAR-RV and HAR-RV-ICS models, respectively; Panels A, B, and C respectively display the 1-step, 5-step, and 22-step predict results for realized volatility. These panels correspond to the performance evaluation of the model within 1-day, 1-week, and1-month predict horizons, respectively.

In summary, through the analysis of the ICS coefficients, we found that most of the coefficients are significantly negative. This indicates that investor climate sentiment (ICS) has a considerable impact on the medium-term and long-term volatility of crude oil futures and may mitigate the volatility of the crude oil futures market. The impact on short-term volatility, however, is relatively weak. The findings of this study are consistent with those of Xu, et al [95]. regarding the impact of climate change on investor behavior and how this behavior influences crude oil prices within the sample.

### HAR model segmented sample analysis

This section divides the sample period into five different stages based on the results of the TOP model between the Investor Climate Sentiment (ICS) index and the realized volatility of crude oil futures (RV^oil^) (**Fig 3**). It further analyzes the specific impact of Investor Climate Sentiment (ICS) on crude oil volatility during the leading or lagging periods within these five stages. The five stages are as follows: the alternating leading phase between RV^oil^ and ICS (from March 27, 2018, to November 29, 2019); the RV^oil^ leading ICS phase (from December 2, 2019, to February 5, 2020); the ICS leading RV^oil^ phase (from February 6, 2020, to June 22, 2020); the RV^oil^ leading ICS phase (from June 23, 2020, to September 7, 2021); and the ICS leading RV^oil^ phase (from September 8, 2021, to December 30, 2022).

The empirical analysis presented in **Table 3** demonstrates that in the phase where ICS and RV^oil^ alternate in leading (Stage One), the estimated coefficient for the monthly ICS index (ICS_m_) is significantly negative over a one-month predictive period. This finding indicates that during this period, Investor Climate Sentiment (ICS) significantly mitigated the long-term volatility in the crude oil futures market.

**Table 3:** HAR model first stage estimation.

Subsample	Variable	Panel A		Panel B		Panel C	
		I	II	I	II	I	II
**2018.3.27–2019.11.29 (Stage One)**	${RV}_{t-1}^{oil}$	0.070	0.141**	0.083	0.083	0.014	0.012
		(1.050)	(2.024)	(1.545)	(1.546)	(0.320)	(0.276)
	${RV}_{w}^{oil}$	0.480***	0.486***	0.440***	0.420***	0.228***	0.197***
		(4.531)	(4.361)	(5.136)	(4.866)	(3.182)	(2.800)
	${RV}_{m}^{oil}$	0.144	0.112	0.095	0.105	0.116	0.113
		(1.287)	(0.939)	(1.032)	(1.134)	(1.512)	(1.504)
	**ICS**_**t−1**_		-0.025		0.110		-0.014
			(-0.126)		(0.713)		(-0.113)
	**ICS**_**w**_		-0.385		-0.382		0.253
			(-0.821)		(-1.052)		(0.855)
	**ICS**_**m**_		-0.730		-0.677		-2.243***
			(-0.837)		(-1.003)		(-4.076)
	**C**	0.096***	0.249*	0.143***	0.292***	0.252***	0.574***
		(2.919)	(1.958)	(5.274)	(2.967)	(11.119)	(7.153)
	**R** ^**2**^	0.237	0.315	0.327	0.337	0.169	0.225

**Note:** T-statistics in parentheses; The asterisks

*, ** and *** denote 10%, 5% and 1% levels of significance, respectively

The empirical analysis results from **Tables 4** and **5** reveal that in the stages where RV leads ICS (Stage 2 and Stage 4), the estimated coefficients for the weekly ICS index (ICS_w_) and the monthly ICS index (ICS_m_) are significantly negative within the one-week predict horizon. However, the level of significance is relatively weak. During periods dominated by the pandemic in terms of market sentiment, climate change, as a long-term and structural issue, was still taken into account by investors and had an impact on their sentiment. This is because even during the pandemic, news reports and events related to climate change continued to occur, and this information could influence investors’ emotions and decision-making [100–103].

**Table 4:** HAR model second stage estimation.

Subsample	Variable	Panel A		Panel B		Panel C	
		I	II	I	II	I	II
**2019.12.2–2020.2.5 (Stage Two)**	${RV}_{t-1}^{oil}$	-0.206	0.087	0.014	-0.022	-0.003	-0.001
		(-0.721)	(0.180)	(0.446)	(-0.580)	(-0.397)	(-0.104)
	${RV}_{w}^{oil}$	0.150	-0.223	-0.085	-0.060	-0.028	-0.039
		(0.293)	(-0.322)	(-1.043)	(-0.877)	(-1.442)	(-1.579)
	${RV}_{m}^{oil}$	-3.167**	-4.824	-2.437***	-3.259***	-0.605***	-0.678**
		(-2.625)	(-1.388)	(-12.261)	(-9.144)	(-12.863)	(-5.255)
	**ICS**_**t−1**_		1.704		-0.102		0.041
			(0.802)		(-0.498)		(0.549)
	**ICS**_**w**_		-3.572		-2.681*		-0.422
			(-0.323)		(-3.149)		(-1.370)
	**ICS**_**m**_		20.332		1.348		0.030
			(0.898)		(0.746)		(0.046)
	**C**	1.358***	-2.676	1.128***	1.572*	0.579***	0.673**
		(3.853)	(-0.424)	(29.823)	(3.080)	(64.712)	(3.643)
	**R** ^**2**^	0.464	0.577	0.985	0.998	0.987	0.996

**Note:** T-statistics in parentheses; The asterisks

*, ** and *** denote 10%, 5% and 1% levels of significance, respectively.

**Table 5:** HAR model fourth stage estimation.

Subsample	Variable	Panel A		Panel B		Panel C	
		I	II	I	II	I	II
**2020.6.23–2021.9.7 (Stage Four)**	${RV}_{t-1}^{oil}$	0.134	0.114	0.159*	0.136*	0.053	0.065
		(1.534)	(1.308)	(1.971)	(1.685)	(1.113)	(1.355)
	${RV}_{w}^{oil}$	0.358***	0.369***	0.090	0.127	-0.030	-0.011
		(2.611)	(2.635)	(0.712)	(0.967)	(-0.399)	(-0.137)
	${RV}_{m}^{oil}$	0.248*	0.231	0.439***	0.347*	0.493***	0.410***
		(1.712)	(1.218)	(3.368)	(1.892)	(6.119)	(3.976)
	**ICS**_**t−1**_		0.316		0.384		-0.088
			(1.089)		(1.465)		(-0.541)
	**ICS**_**w**_		0.215		0.094		-0.351
			(0.466)		(0.211)		(-1.276)
	**ICS**_**m**_		-0.700		-0.830*		0.243
			(-1.490)		(-1.676)		(0.890)
	**C**	0.166*	0.185	0.266***	0.345***	0.431***	0.474***
		(1.960)	(1.552)	(3.519)	(3.005)	(8.650)	(6.917)
	**R** ^**2**^	2.252	0.268	0.320	0.348	0.331	0.353

**Note:** T-statistics in parentheses; The asterisks

*, ** and *** denote 10%, 5% and 1% levels of significance, respectively.

**Tables 6** and **7** present the estimation results of the HAR model when ICS guides RV (Stages 3 and 5). It can be observed from **Table 6** that the regression coefficient of the monthly ICS index (ICS_m_) is significantly negative within the predict horizons of 1 day, 1 week, and 1 month. In **Table 7**, the regression coefficient for the monthly ICS index (ICS_m_) also exhibits a significant negative correlation at the 1-day and 1-week predict levels. Concurrently, the regression coefficient for the weekly ICS index (ICS_m_) similarly shows a significant negative value within the 1-month predict horizon. The results indicate that when investor climate sentiment (ICS) is in the leading phase, ICS will impact the crude oil market volatility in the medium and long term.

**Table 6:** HAR model third stage estimation.

Subsample	Variable	Panel A		Panel B		Panel C	
		I	II	I	II	I	II
**2020.2.6–2020.6.22 (Stage Three)**	${RV}_{t-1}^{oil}$	0.149	0.140	0.119	0.105	0.037	0.032
		(0.955)	(0.907)	(1.062)	(1.407)	(0.421)	(1.115)
	${RV}_{w}^{oil}$	0.543**	0.230	0.488***	-0.137	0.009	-0.161***
		(2.442)	(0.871)	(3.033)	(-1.072)	(0.066)	(-3.426)
	${RV}_{m}^{oil}$	-0.272	-0.239	-0.595***	-0.502***	-0.396**	-0.178***
		(-1.077)	(-0.931)	(-3.267)	(-4.033)	(-2.693)	(-3.573)
	**ICS**_**t−1**_		-0.046		0.470		0.150
			(-0.033)		(0.695)		(0.596)
	**ICS**_**w**_		4.306		6.238***		-0.360
			(0.917)		(2.740)		(-0.401)
	**ICS**_**m**_		-14.256*		-26.820***		-22.022***
			(-1.938)		(-7.521)		(-11.729)
	**C**	0.788*	2.487***	1.628***	4.997***	2.306***	4.780***
		(1.951)	(2.823)	(5.571)	(11.700)	(8.293)	(26.050)
	**R** ^**2**^	0.237	0.296	0.326	0.720	0.164	0.918

**Note:** T-statistics in parentheses; The asterisks

*, ** and *** denote 10%, 5% and 1% levels of significance, respectively.

**Table 7:** HAR model fifth stage estimation.

Subsample	Variable	Panel A		Panel B		Panel C	
		I	II	I	II	I	II
**2021.9.8–2022.12.30 (Stage Five)**	${RV}_{t-1}^{oil}$	0.138***	0.131***	0.076**	0.073**	0.032	0.038
		(2.737)	(2.602)	(2.034)	(1.981)	(1.004)	(1.268)
	${RV}_{w}^{oil}$	0.308***	0.316***	0.360***	0.374***	0.198***	0.204***
		(3.848)	(3.947)	(6.103)	(6.415)	(3.953)	(4.251)
	${RV}_{m}^{oil}$	0.282***	0.205**	0.222***	0.079	0.231***	0.044
		(3.345)	(2.121)	(3.574)	(1.125)	(4.357)	(0.761)
	**ICS**_**t−1**_		0.142		0.270*		-0.063
			(0.638)		(1.664)		(-0.473)
	**ICS**_**w**_		0.240		-0.290		-0.738***
			(0.675)		(-1.112)		(-3.471)
	**ICS**_**m**_		-0.776**		-0.649**		-0.062
			(-2.244)		(-2.568)		(-0.302)
	**C**	0.155***	0.237***	0.235***	0.376***	0.388***	0.572***
		(3.644)	(3.675)	(7.481)	(7.983)	(14.566)	(14.858)
	**R** ^**2**^	0.243	0.251	0.328	0.351	0.236	0.308

**Note:** T-statistics in parentheses; The asterisks

*, ** and *** denote 10%, 5% and 1% levels of significance, respectively.

It is worth noting that in this phase of the study, the significance of the regression coefficients for investor climate sentiment has significantly improved compared to the other three phases, with the majority of the coefficients being significant at the 1% and 5% significance levels. This finding indicates that when ICS guides RV, investors pay more attention to the risks brought about by climate change. This attention prompts investors to maintain a more calm and rational attitude in the face of market volatility. According to the research by Li, et al. [104] and Ding, et al. [105], when investors’ attention to and sentiment about climate change align, this consensus tends to reduce market uncertainty, thereby mitigating market volatility.

In conclusion, the estimation results of the HAR model with segmented samples reveal that investor climate sentiment (ICS) exerts a significant dampening effect on the long-term volatility of the crude oil market, regardless of whether it plays a dominant role. This finding is consistent with the analysis results of the full-sample model. The difference lies in that, after the sample segmentation, when ICS guides RV, the inhibitory effect of ICS on the medium to long-term volatility in the crude oil market is significantly enhanced. In contrast, the full-sample analysis failed to reveal the trend in the intensity of the impact of investor climate sentiment (ICS) on crude oil futures volatility (RV) as the data structure undergoes dynamic changes.

### Out-of-sample volatility predicting based on the HAR model

Compared to the results within the sample, decision-makers and investors usually pay more attention to the predictive performance of the model [106–110]. This is because they hope to use the model to accurately predict future volatility, enabling them to make more informed decisions. In light of this, the research objectives of this section are also divided into two levels: First, to investigate whether incorporating Investor Climate Sentiment (ICS) into the volatility model can enhance its predictive capability for out-of-sample data; Secondly, this paper will analyze whether using the Thermal Optimal Path (TOP) method to segment the sample can further improve the prediction accuracy of the model compared to full-sample predicting. To comprehensively assess the model’s predictive performance in the crude oil market, this section utilizes three loss functions: Mean Squared Error (MSE), Root Mean Squared Error (RMSE), and Mean Absolute Error (MAE). These loss functions provide effective tools for quantitatively evaluating the accuracy of predictions.


$$MSE=N^{-1}\sum_{t=1}^{N}{\left({RV}_{t}^{P}-{RV}_{t}\right)}^{2}$$ (12)

$$RMSE=\sqrt{N^{-1}\sum_{t=1}^{N}{\left({RV}_{t}^{P}-{RV}_{t}\right)}^{2}}$$ (13)

$$MAE=N^{-1}\sum_{t=1}^{N}\left|{RV}_{t}^{P}-{RV}_{t}\right|$$ (14)

Where, ${RV}_{t}^{P}$ is the predicted value of realized volatility of crude oil futures; RV_t_ is the true value of realized volatility of crude oil futures, and N is the predict days.

### Predictive performance of the model out-of-sample in the case of full sample

**Table 8** reveals the impact of incorporating Investor Climate Sentiment (ICS) on the out-of-sample predict of crude oil futures volatility (RV^oil^). In the 1-day time scale predict of realized volatility (RV^oil^), the introduction of ICS led to a 1% decrease in Mean Squared Error (MSE), a 0.3% reduction in Root Mean Squared Error (RMSE), and a 3.1% increase in R-squared. These results suggest that the additional information gained from incorporating investor climate sentiment (ICS) may have a positive effect on the prediction of volatility in the short term. However, in terms of long-term predicting, the introduction of ICS does not demonstrate a significant predictive advantage. This could be because long-term predicts span multiple distinct market cycles, and the variety and volatility of these market cycles may diminish the influence of Investor Climate Sentiment (ICS) on the prediction of crude oil futures volatility.

**Table 8:** Performance of the HAR model in predicting crude oil futures volatility with and without ICS in full-sample settings.

Subsample	Models	MSE	RMSE	MAE	R-Square
**2018.3.27–2022.12.30 (Full Sample)**	**Panel A**	0.089	0.298	0.193	0.161
	**Panel B**	0.014	0.116	0.079	0.786
	**Panel C**	0.010	0.101	0.080	0.730
	**Panel A-ICS**	**0.088**	**0.297**	**0.193**	**0.166**
	**Panel B-ICS**	0.0140	0.118	0.081	0.779
	**Panel C-ICS**	0.011	0.105	0.085	0.708

**Note:** Panels A, B, and C represent the volatility predicts for 1-day, 1-week, and 1-month time horizons, respectively, and are primarily used to evaluate the model’s performance in predicting short-term, medium-term, and long-term volatility; Panels A-ICS, B-ICS, and C-ICS respectively display the volatility predicts over 1-day, 1-week, and 1-month horizons after incorporating investor climate sentiment factors. These panels are primarily used to assess the performance of the model that includes investor climate sentiment in predicting short-term, medium-term, and long-term volatility; The trend of the prediction results is detailed in **Fig 4**.

**Fig 4:**
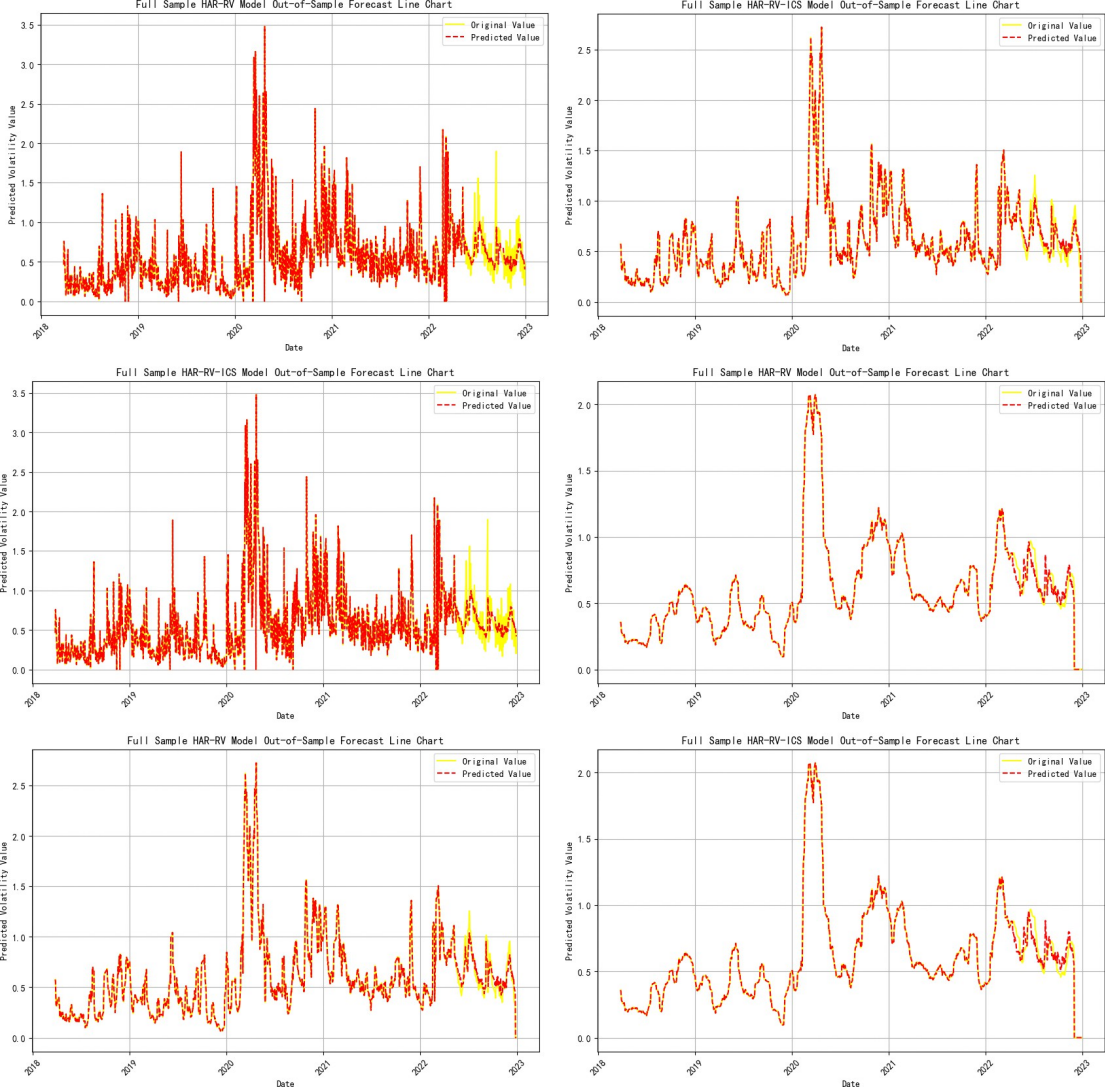
Trend of out-of-sample volatility predict for crude oil futures (full sample). **Note:** The yellow line represents the actual realized volatility, and the red line represents the predicted realized volatility; (a), (c), and (e) represent the volatility forecasts for 1-day, 1-week, and 1-month time horizons, respectively; (b), (d), and (f) respectively show the trend of volatility predictions for 1-day, 1-week, and 1-month horizons after incorporating investor climate sentiment factors.

### Out-of-sample prediction performance of the model in the split sample scenario

In this section, following the empirical analysis results of the TOP model (**Fig 3**), the same methodology is applied to segment the sample of realized volatility for crude oil futures, and subsequent volatility forecasting is conducted. This division is based on the stages where Investor Climate Sentiment (ICS) guides the realized volatility of crude oil (RV^oil^).

**Table 9** presents the out-of-sample prediction performance of each model after sample segmentation. During the phase of interaction between Investor Climate Sentiment (ICS) and crude oil futures volatility (RV^oil^) (Stage One), the prediction error for RV^oil^ at the 1-month time scale significantly decreased, with a 16.7% reduction in MSE, a 2.6% decrease in RMSE, a 14.3% drop in MAE, and a 1.3% increase in the model’s R-squared.

**Table 9:** The prediction performance of the HAR model based on the Top method segmentation for crude oil futures volatility with/without ICS at different time intervals.

Subsample	Models	MSE	RMSE	MAE	R-Square
**2018.3.27–2019.11.29 (Stage One)**	**Panel A**	0.040	0.200	0.041	0.831
	**Panel B**	0.006	0.079	0.021	0.825
	**Panel C**	0.006	0.076	0.021	0.766
	**Panel A-ICS**	0.040	0.200	0.042	0.931
	**Panel B-ICS**	0.006	0.080	0.021	0.821
	**Panel C-ICS**	**0.005**	**0.074**	**0.018**	**0.776**
**2019.12.2–2020.2.5 (Stage Two)**	**Panel A**	0.031	0.177	0.050	0.650
	**Panel B**	0.009	0.094	0.031	0.835
	**Panel C**	0.010	0.098	0.057	0.336
	**Panel A-ICS**	0.202	0.450	0.148	0.254
	**Panel B-ICS**	0.012	0.108	0.036	0.778
	**Panel C-ICS**	**0.007**	**0.086**	**0.049**	**0.495**
**2020.2.6–2020.6.22 (Stage Three)**	**Panel A**	0.021	0.145	0.040	0.969
	**Panel B**	0.062	0.251	0.077	0.889
	**Panel C**	0.186	0.431	0.197	0.698
	**Panel A-ICS**	**0.006**	**0.078**	**0.019**	**0.991**
	**Panel B-ICS**	**0.015**	**0.122**	**0.034**	**0.974**
	**Panel C-ICS**	**0.144**	**0.380**	**0.152**	**0.765**
**2020.6.23–2021.9.7 (Stage Four)**	**Panel A**	0.009	0.093	0.040	0.931
	**Panel B**	0.010	0.103	0.059	0.880
	**Panel C**	0.011	0.106	0.062	0.871
	**Panel A-ICS**	**0.008**	**0.091**	**0.039**	**0.934**
	**Panel B-ICS**	0.010	0.104	0.061	0.876
	**Panel C-ICS**	**0.010**	**0.100**	**0.058**	**0.887**
**2021.9.8–2022.12.30 (Stage Five)**	**Panel A**	0.002	0.048	0.009	0.979
	**Panel B**	0.001	0.026	0.004	0.990
	**Panel C**	0.001	0.015	0.003	0.996
	**Panel A-ICS**	0.002	**0.047**	0.009	**0.980**
	**Panel B-ICS**	0.001	**0.024**	0.004	**0.991**
	**Panel C-ICS**	0.001	**0.013**	**0.002**	**0.997**

**Note:** Panels A, B, and C represent the volatility predicts for 1-day, 1-week, and 1-month time horizons, respectively, and are primarily used to evaluate the model’s performance in predicting short-term, medium-term, and long-term volatility; Panels A-ICS, B-ICS, and C-ICS respectively display the volatility predicts over 1-day, 1-week, and 1-month horizons after incorporating investor climate sentiment factors. These panels are primarily used to assess the performance of the model that includes investor climate sentiment (ICS) in predicting short-term, medium-term, and long-term volatility; The trend of the prediction results is detailed in **Figs 5–9**.

**Fig 5:**
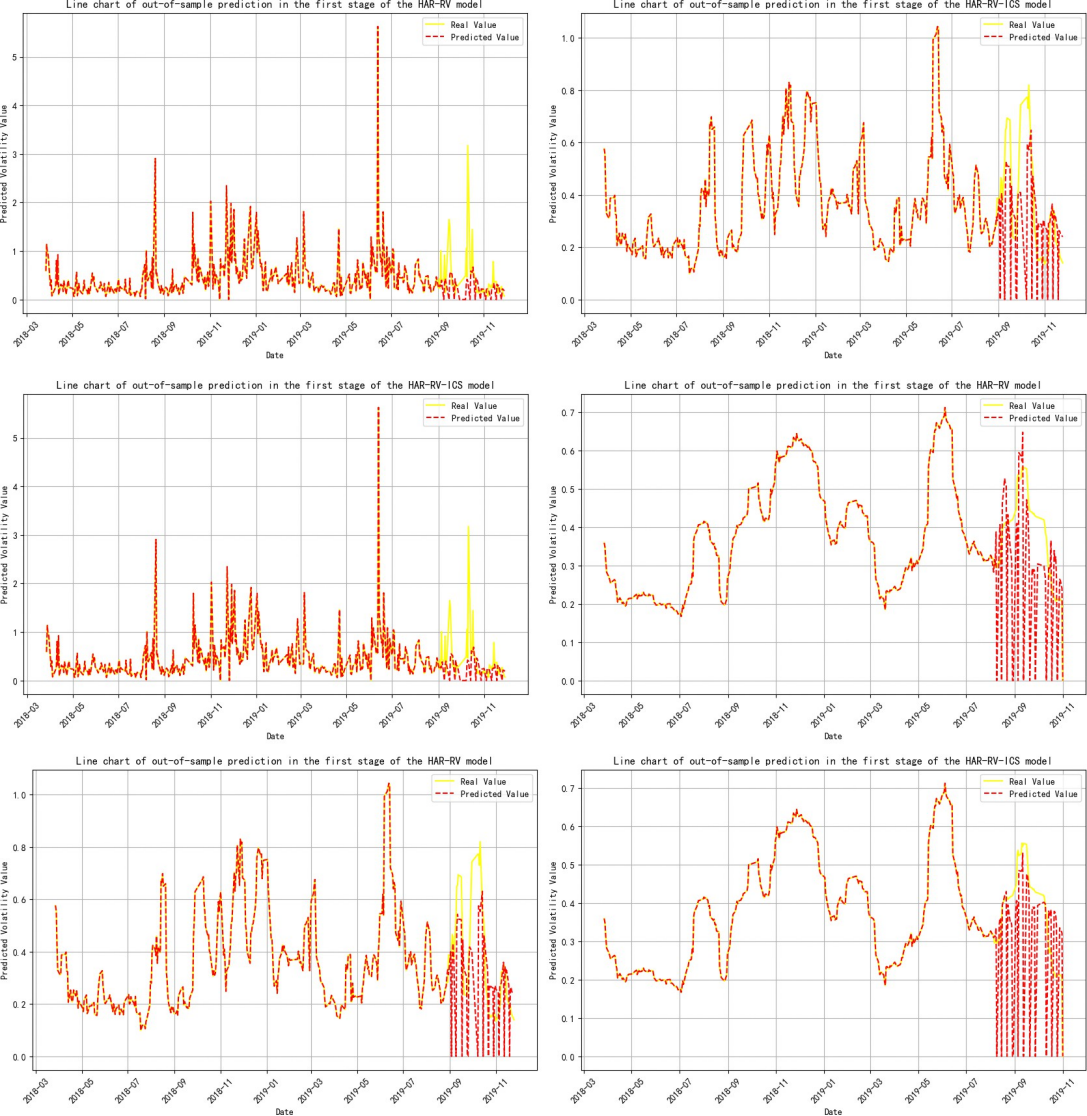
Trend of out-of-sample volatility predict for crude oil futures (Stage One). **Note:** The yellow line represents the actual realized volatility, and the red line represents the predicted realized volatility; (a), (c), and (e) represent the volatility forecasts for 1-day, 1-week, and 1-month time horizons, respectively; (b), (d), and (f) respectively show the trend of volatility predictions for 1-day, 1-week, and 1-month horizons after incorporating investor climate sentiment factors.

**Fig 6:**
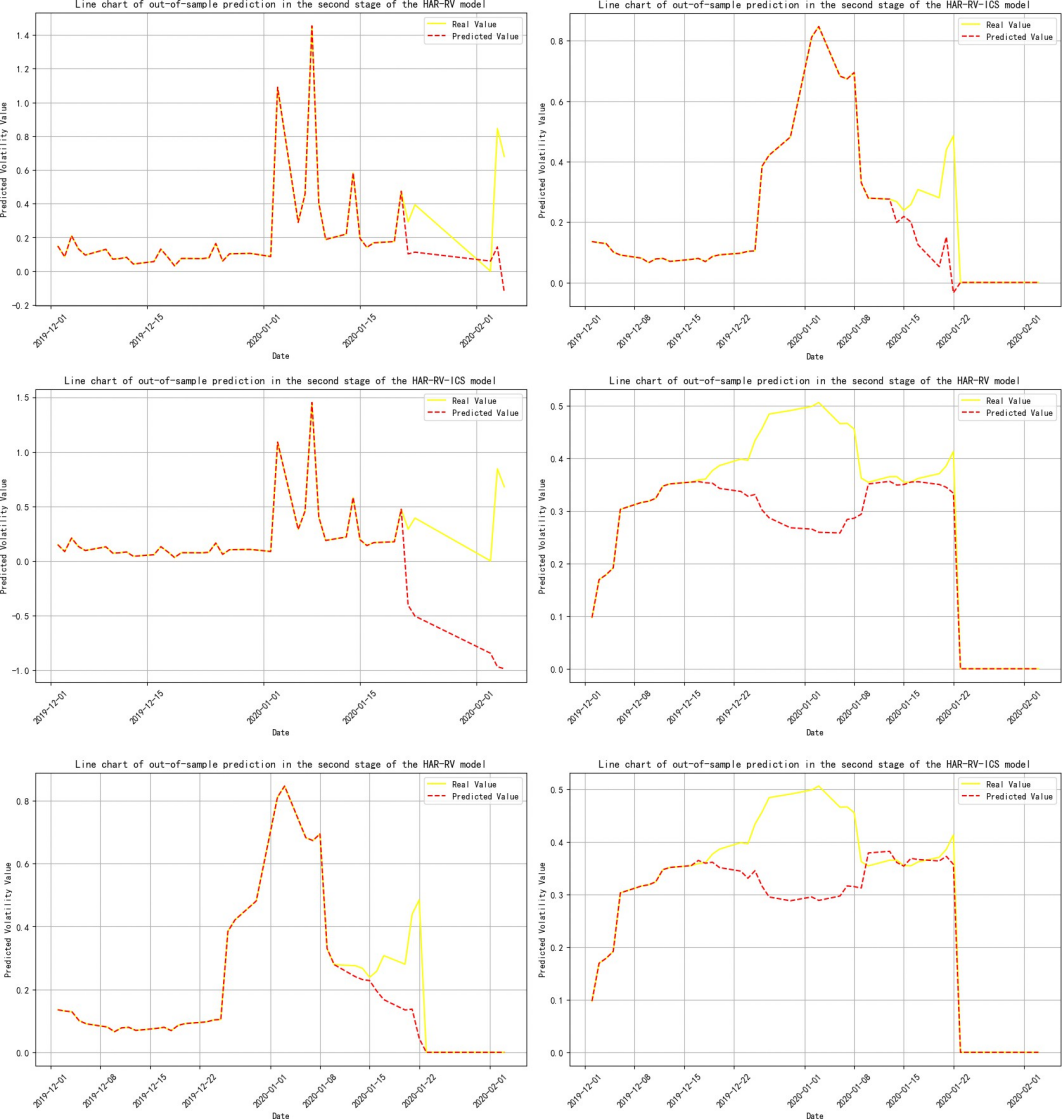
Trend of out-of-sample volatility predict for crude oil futures (Stage Two). **Note:** The yellow line represents the actual realized volatility, and the red line represents the predicted realized volatility.

**Fig 7:**
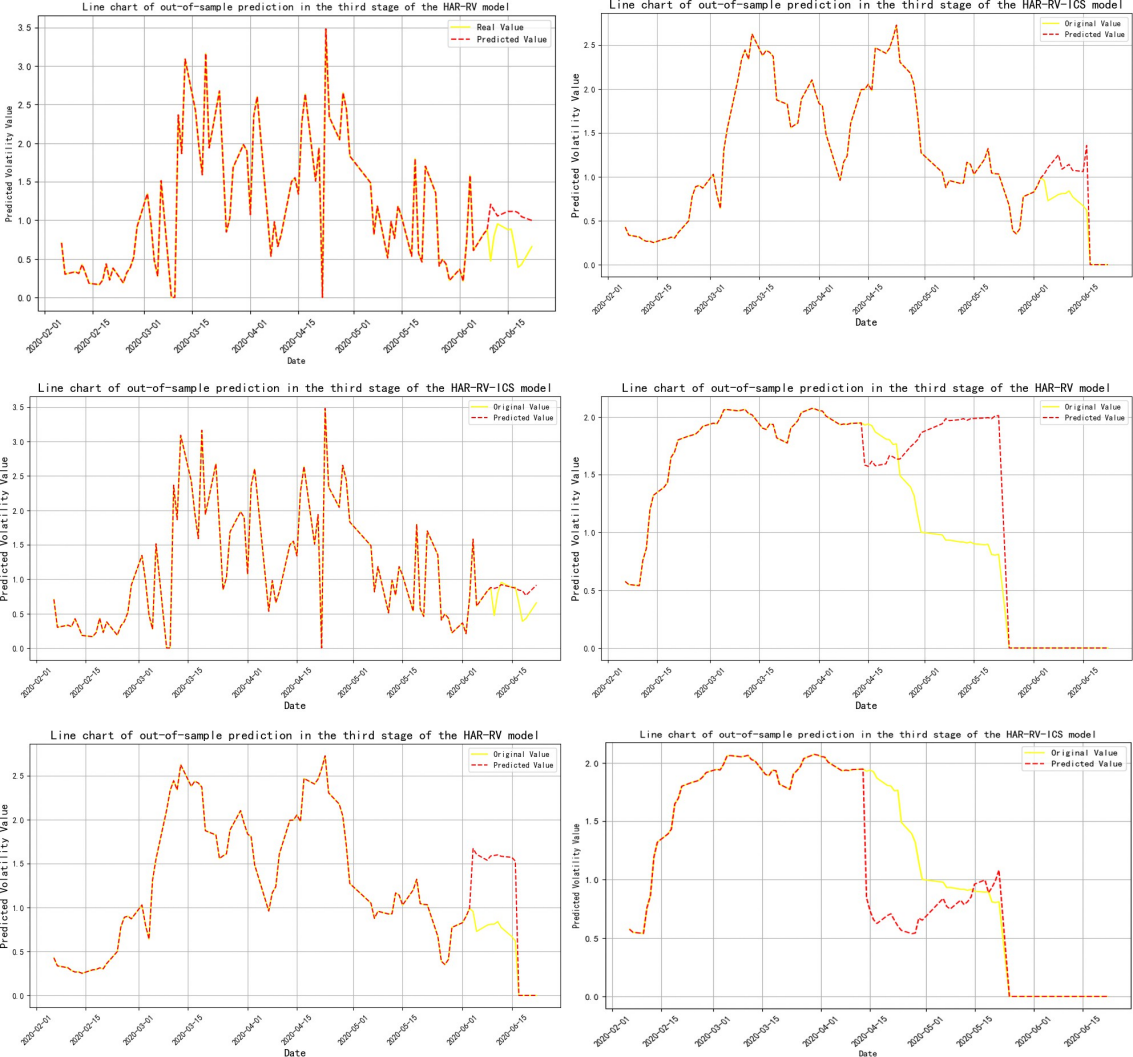
Trend of out-of-sample volatility predict for crude oil futures (Stage Three). **Note:** The yellow line represents the actual realized volatility, and the red line represents the predicted realized volatility.

**Fig 8:**
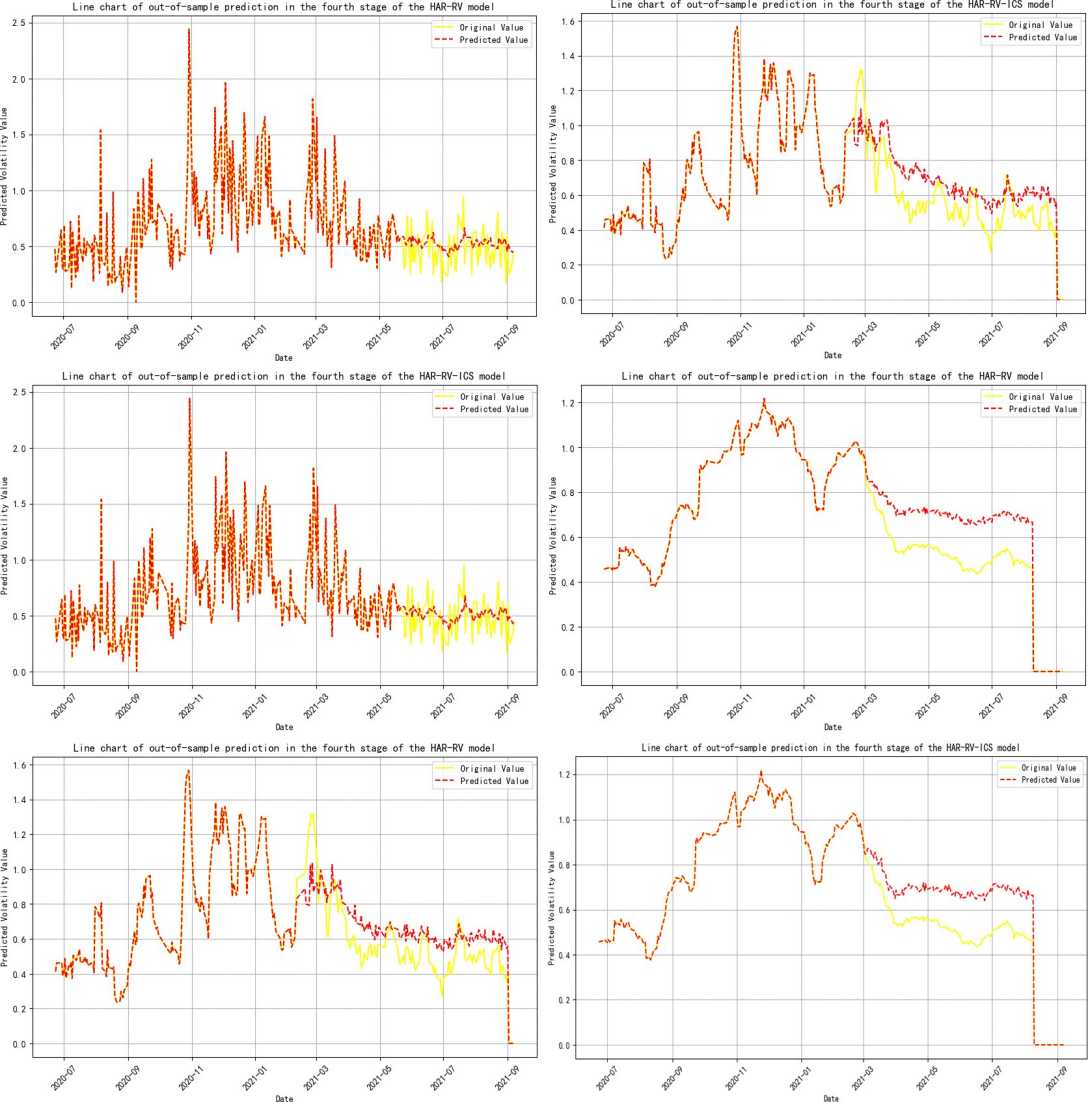
Trend of out-of-sample volatility predict for crude oil futures (Stage Four). **Note:** The yellow line represents the actual realized volatility, and the red line represents the predicted realized volatility.

**Fig 9:**
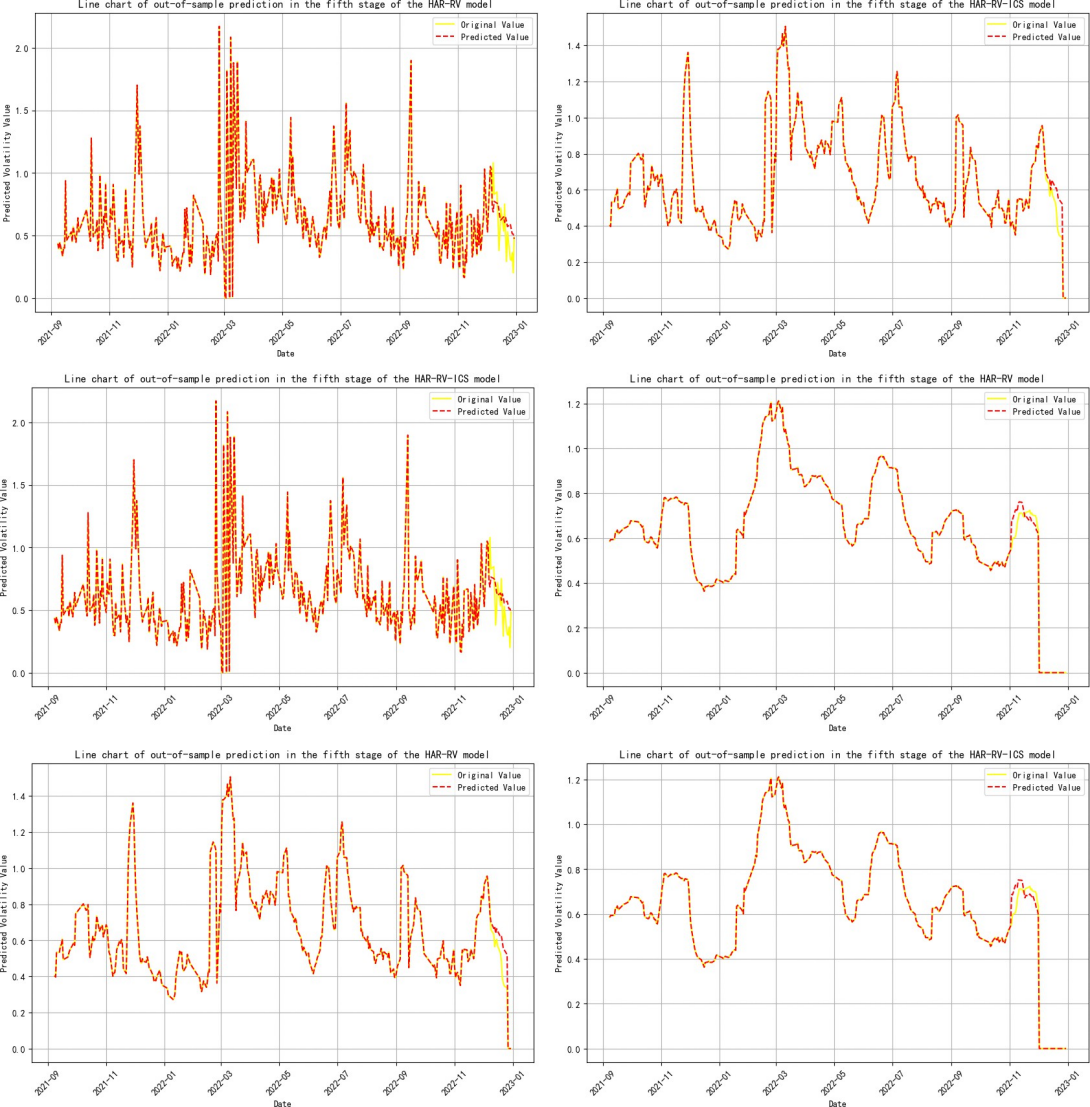
Trend of out-of-sample volatility predict for crude oil futures (Stage Five). **Note:** The yellow line represents the actual realized volatility, and the red line represents the predicted realized volatility.

During the phases where crude oil futures volatility (RV^oil^) guides Investor Climate Sentiment (ICS) (Stages 2 and 4), the prediction errors for RV^oil^ at the 1-day and 1-month time scales have notably decreased, although the extent of the reduction is not significant.

In the three-stage model prediction of ICS guiding crude oil futures volatility (stages 2, 3, and 5), the model prediction results for stage 2 show that the prediction error of RV is significantly reduced on a 1-month time scale. Specifically, the MSE was reduced by 30%, the mean RMSE decreased by 12.24%, the MAE was reduced by 14.04%, and the model’s R-squared also increased by 1.3%. In the prediction results of Stage 3 and Stage 5, the prediction error of RV shows a significant downward trend at all time scales, whether it is 1-day, 1-week, or 1-month.This phenomenon further confirms that when investor climate sentiment (ICS) becomes a key factor influencing the volatility of crude oil futures (RV^oil^), ICS plays a significant role as a leading indicator, revealing the market’s expectations for future oil price volatility in advance.

Based on the comprehensive analysis above, this section compares the model prediction effects before and after sample segmentation. From this, the following conclusion can be drawn: the introduction of Investor Climate Sentiment (ICS) significantly improves the predictive accuracy of the model, whether it is full-sample model prediction or model prediction after sample segmentation.

This indicates that ICS contains a substantial amount of additional information about realized volatility. Therefore, incorporating ICS into the out-of-sample predicts of the model will help optimize the predictive performance of traditional HAR models. This finding aligns with the conclusions of Liu, et al. [38] and Liu, et al. [111] in their studies on the impact of investor behavior on the prediction of volatility in energy markets. Additionally, by comparing the full-sample analysis, we find that the volatility prediction results after sample segmentation were different. During the phase where Investor Climate Sentiment (ICS) plays a guiding role, its predictive capability for short-term, medium-term, and long-term volatility has significantly improved. Further analysis indicates that even when ICS is in a lagging or mutually guiding phase, it still effectively reduces the prediction error of the model on a 1-month time scale. Therefore, employing the Thermal Optimal Path (TOP) method to segment the sample can more accurately uncover the impact of the ICS on the precision of crude oil volatility forecasts as the data structure of the variables changes.

## Conclusions

This paper, through empirical analysis of the impact of investor climate sentiment (ICS) on crude oil futures volatility (RV^oil^), reveals the significant role of investor climate sentiment in the formation process of asset prices. As a major developing country, the development and changes in China’s crude oil market are influenced by a variety of domestic and international factors, making the study of the impact of climate change on China’s crude oil market more complex. Compared to the macro factors of climate change affecting the crude oil market, this paper places more emphasis on a micro-level analysis. This study primarily investigates whether the impact of climate change on investment sentiment plays a significant role in the predictability of fluctuations in China’s crude oil market. The following is a summary of the research findings:

Firstly, by using the Thermal Optimal Path (TOP) method, we found that the crude oil futures volatility (RV^oil^) had a strong guiding effect on investor sentiment (ICS) in the early stage of epidemic. Over time, Investor Climate Sentiment (ICS) has increasingly become a key factor guiding the volatility of crude oil futures (RV^oil^), with its dominance and influence both showing an upward trend. This change underscores the alteration in investor sentiment towards pandemic and climate change issues. Simultaneously, it indicates that market participants’ focus on and emotional response to climate change is progressively strengthening.

Second, based on the lead-lag relationship between ICS and RV^oil^, this study divides the sample period into five stages and compares the estimation results of the model before and after the segmentation. During the phase where ICS and RV^oil^ mutually guide each other (March 27, 2018—November 29, 2019) and the phases where RV^oil^ dominates ICS (December 2, 2019—February 5, 2020, June 23, 2020—September 7, 2021), ICS has an inhibitory effect on the long-term volatility of the crude oil market. In the phases where ICS dominates RV^oil^ (February 6, 2020—June 22, 2020, September 8, 2021—December 30, 2022), it is shown that ICS also has a similar inhibitory effect on medium to long-term volatility. Compared to the full-sample model, this paper segments the data sample based on the Thermal Optimal Path (TOP) method, revealing that the impact period and the effect of ICS on crude oil volatility (RV^oil^) are enhanced during the guiding phase.

Third, incorporating investor climate sentiment (ICS) into the in-sample estimation of the HAR model significantly enhances the model’ s goodness of fit. In the out-of-sample prediction of crude oil volatility, the integration of ICS significantly reduces the prediction error, indicating that ICS, as an important variable for predicting crude oil futures volatility, provides additional predictive information.

Fourth, by comparing the out-of-sample prediction results before and after the sample segmentation, this study find that ICS significantly enhances the predictive capability for crude oil market volatility during the guiding phase. Furthermore, even in the non-guiding phase, ICS is able to effectively reduce the one-month predicting error, highlighting the advantage of the TOP method in revealing the impact of ICS on volatility prediction.

In summary, the following policy recommendations are provided in this paper:

First, given the increasingly prominent role of investor climate sentiment (ICS) in guiding crude oil futures prices and volatility, it is recommended that the government and relevant departments intensify the propaganda and education on climate change knowledge for investors. This will enhance market participants’ understanding of how climate change affects the crude oil market mechanism. The purpose of such propaganda and education is to enhance the stability and predictability of the market, thereby providing a more stable and transparent investment environment for market participants.

Second, based on the lead-lag relationship between ICS and crude oil volatility (RV^oil^), it is recommended that the regulatory authorities establish a dynamic monitoring system to track changes in investor climate sentiment in real-time. During the critical periods when ICS leads RV^oil^, market volatility alerts should be issued in a timely manner to assist investors and policymakers in making more scientifically sound investment and regulatory decisions.

Third, considering that ICS significantly improves the accuracy of predicting crude oil market volatility during the guiding phase, it is recommended that financial regulatory authorities and market participants incorporate ICS as a key variable into volatility prediction models. This approach can enhance the precision of predictions and reduce the uncertainty of investments.

Fourth, it is recommended that relevant research institutions and financial organizations fully consider the important factor of investor climate sentiment when constructing crude oil volatility prediction models. This can provide market participants with more comprehensive and accurate predicting information, thereby offering strong support for their decision-making.

Therefore, using measures such as the ICS index to track investor sentiment and expectations can more effectively assess and manage the risks brought about by climate change, thereby promoting the stable development of the market. This will contribute to enhancing the market’s resilience against risks and provide robust support for the sustainable development of the economy.

## Supporting information

S1 Data Data set used for empirical analysis. (XLSX)

## References

[1] Ergen I, Rizvanoghlu I. Asymmetric impacts of fundamentals on the natural gas futures volatility: An augmented GARCH approach. Energy Economics. 2016;56:64–74.

[2] Gong X, Ye X, Zhang W, Zhang Y. Predicting energy futures high-frequency volatility using technical indicators: The role of interaction. Energy Economics. 2023;119:106533.

[3] Alberola E, Chevallier J, Chèze Bt. Price drivers and structural breaks in European carbon prices 2005–2007. Energy policy. 2008;36(2):787–97.

[4] Xie Q, Hao J, Li J, Zheng X. Carbon price prediction considering climate change: A text-based framework. Economic Analysis and Policy. 2022;74:382–401.

[5] Tumala MM, Salisu A, Nmadu YB. Climate change and fossil fuel prices: A GARCH-MIDAS analysis. Energy Economics. 2023;124:106792.

[6] Charles A, Darné O. Forecasting crude-oil market volatility: Further evidence with jumps. Energy Economics. 2017;67:508–19.

[7] Lin B, Wesseh PK Jr, Appiah MO. Oil price fluctuation, volatility spillover and the Ghanaian equity market: Implication for portfolio management and hedging effectiveness. Energy Economics. 2014;42:172–82.

[8] Wang Y, Liu L, Ma F, Wu C. What the investors need to know about forecasting oil futures return volatility. Energy Economics. 2016;57:128–39.

[9] Wen F, Xiao J, Huang C, Xia X. Interaction between oil and US dollar exchange rate: nonlinear causality, time-varying influence and structural breaks in volatility. Applied Economics. 2018;50(3):319–34.

[10] Bahloul W, Bouri A. The impact of investor sentiment on returns and conditional volatility in US futures markets. Journal of Multinational Financial Management. 2016;36:89–102.

[11] Chen R, Xu J. Forecasting volatility and correlation between oil and gold prices using a novel multivariate GAS model. Energy Economics. 2019;78:379–91.

[12] Deeney P, Cummins M, Dowling M, Bermingham A. Sentiment in oil markets. International Review of Financial Analysis. 2015;39:179–85.

[13] Cunado J, De Gracia FP. Oil prices, economic activity and inflation: evidence for some Asian countries. The Quarterly Review of Economics and Finance. 2005;45(1):65–83.

[14] Cunado J, Jo S, de Gracia FP. Macroeconomic impacts of oil price shocks in Asian economies. Energy Policy. 2015;86:867–79.

[15] Sornette D, Zhou W-X. Non-parametric determination of real-time lag structure between two time series: the ‘optimal thermal causal path’method. Quantitative Finance. 2005;5(6):577–91.

[16] Batten JA, Maddox GE, Young MR. Does weather, or energy prices, affect carbon prices? Energy Economics. 2021;96:105016.

[17] Zheng Y, Zhou M, Wen F. Asymmetric effects of oil shocks on carbon allowance price: evidence from China. Energy Economics. 2021;97:105183.

[18] Shi C, Zeng Q, Zhi J, Na X, Cheng S. A study on the response of carbon emission rights price to energy price macroeconomy and weather conditions. Environmental Science and Pollution Research. 2023;30(12):33833–48. doi: 10.1007/s11356-022-24577-2 36502476 PMC9741706

[19] Dong J, Dai W, Li J. Exploring the linear and nonlinear causality between internet big data and stock markets. Journal of Systems Science and Complexity. 2020;33(3):783–98.

[20] Yang Y-H, Shao Y-H. Time-dependent lead-lag relationships between the VIX and VIX futures markets. The North American Journal of Economics and Finance. 2020;53:101196.

[21] Yao C-Z, Li H-Y. Time-varying lead–lag structure between investor sentiment and stock market. The North American Journal of Economics and Finance. 2020;52:101148.

[22] Chen R, Yu J, Jin C, Bao W. Internet finance investor sentiment and return comovement. Pacific-Basin Finance Journal. 2019;56:151–61.

[23] Chiang IHE, Hughen WK, Sagi JS. Estimating oil risk factors using information from equity and derivatives markets. The Journal of Finance. 2015;70(2):769–804.

[24] Gong X, Lin B. The incremental information content of investor fear gauge for volatility forecasting in the crude oil futures market. Energy Economics. 2018;74:370–86.

[25] Wang Y, Wei Y, Wu C, Yin L. Oil and the short-term predictability of stock return volatility. Journal of Empirical Finance. 2018;47:90–104.

[26] Xu B, Ouenniche J. A data envelopment analysis-based framework for the relative performance evaluation of competing crude oil prices’ volatility forecasting models. Energy Economics. 2012;34(2):576–83.

[27] Andersen TG, Bollerslev T, Diebold FX, Labys P. Modeling and forecasting realized volatility. Econometrica. 2003;71(2):579–625.

[28] Andersen TG, Bollerslev T, Meddahi N. Analytical evaluation of volatility forecasts. International Economic Review. 2004;45(4):1079–110.

[29] Lyócsa Š, Molnár P. Exploiting dependence: Day-ahead volatility forecasting for crude oil and natural gas exchange-traded funds. Energy. 2018;155:462–73.

[30] Wen F, Zhao Y, Zhang M, Hu C. Forecasting realized volatility of crude oil futures with equity market uncertainty. Applied Economics. 2019;51(59):6411–27.

[31] Zhu X-h, Zhang H-w, Zhong M-r. Volatility forecasting in Chinese nonferrous metals futures market. Transactions of Nonferrous Metals Society of China. 2017;27(5):1206–14.

[32] Baker M, Wurgler J. Investor sentiment and the cross‐section of stock returns. The journal of Finance. 2006;61(4):1645–80.

[33] Campbell JY, Grossman SJ, Wang J. Trading volume and serial correlation in stock returns. The Quarterly Journal of Economics. 1993;108(4):905–39.

[34] Changsheng H, Yongfeng W. Investor sentiment and assets valuation. Systems Engineering Procedia. 2012;3:166–71.

[35] De Long JB, Shleifer A, Summers LH, Waldmann RJ. Noise trader risk in financial markets. Journal of political Economy. 1990;98(4):703–38.

[36] Kumar A, Lee CM. Retail investor sentiment and return comovements. The Journal of Finance. 2006;61(5):2451–86.

[37] Lechthaler F, Leinert L. Moody oil: What is driving the crude oil price? Empirical Economics. 2019;57(5):1547–78.

[38] Liu Y, Niu Z, Suleman MT, Yin L, Zhang H. Forecasting the volatility of crude oil futures: The role of oil investor attention and its regime switching characteristics under a high-frequency framework. Energy. 2022;238:121779.

[39] Chen R, Bao W, Jin C. Investor sentiment and predictability for volatility on energy futures Markets: Evidence from China. International Review of Economics & Finance. 2021;75:112–29.

[40] Chiu C-wJ Harris RD, Stoja E, Chin M. Financial market volatility, macroeconomic fundamentals and investor sentiment. Journal of Banking & Finance. 2018;92:130–45.

[41] Du D, Gunderson RJ, Zhao X. Investor sentiment and oil prices. Journal of Asset Management. 2016;17:73–88.

[42] Qadan M, Nama H. Investor sentiment and the price of oil. energy economics. 2018;69:42–58.

[43] Andrei D, Hasler M. Investor attention and stock market volatility. The review of financial studies. 2015;28(1):33–72.

[44] Ben-Rephael A, Da Z, Israelsen RD. It depends on where you search: Institutional investor attention and underreaction to news. The Review of Financial Studies. 2017;30(9):3009–47.

[45] Pham L, Huynh TLD. How does investor attention influence the green bond market? Finance Research Letters. 2020;35:101533.

[46] Wang H, Xu L, Sharma SS. Does investor attention increase stock market volatility during the COVID-19 pandemic? Pacific-Basin Finance Journal. 2021;69:101638.

[47] Gong X, Lai P, He M, Wen D. Climate risk and energy futures high frequency volatility prediction. Energy. 2024;307:132466.

[48] Allen F, Babus A, Carletti E. Asset commonality, debt maturity and systemic risk. Journal of Financial Economics. 2012;104(3):519–34.

[49] Cardarelli R, Elekdag S, Lall S. Financial stress and economic contractions. Journal of financial Stability. 2011;7(2):78–97.

[50] Fontana G, Sawyer M. Towards post-Keynesian ecological macroeconomics. Ecological Economics. 2016;121:186–95.

[51] Nordhaus WD, Yang Z. A regional dynamic general-equilibrium model of alternative climate-change strategies. The American Economic Review. 1996:741–65.

[52] Cavallo E, Galiani S, Noy I, Pantano J. Catastrophic natural disasters and economic growth. Review of Economics and Statistics. 2013;95(5):1549–61.

[53] Klomp J. Financial fragility and natural disasters: An empirical analysis. Journal of Financial stability. 2014;13:180–92.

[54] Murshed S, Paull DJ, Griffin AL, Islam MA. A parsimonious approach to mapping climate-change-related composite disaster risk at the local scale in coastal Bangladesh. International journal of disaster risk reduction. 2021;55:102049.

[55] Nand MM, Bardsley DK. Climate change loss and damage policy implications for Pacific Island Countries. Local Environment. 2020;25(9):725–40.

[56] Noth F, Schüwer U. Natural disasters and bank stability: Evidence from the US financial system. Journal of Environmental Economics and Management. 2023;119:102792.

[57] Strobl E. The economic growth impact of hurricanes: Evidence from US coastal counties. Review of Economics and Statistics. 2011;93(2):575–89.

[58] Coffel ED, Mankin JS. Thermal power generation is disadvantaged in a warming world. Environmental Research Letters. 2021;16(2):024043.

[59] Mo J, Duan H, Fan Y, Wang S. China’s energy and climate targets in the paris agreement: integrated assessment and policy options. Econ Res J. 2018;53(09):168–81.

[60] ZHU S, LU X, YANG J, Jing C, SHE Y, CHEN Y-b, WANG W. Study on the Physical Risk of the Impact of Climate Change on Economic and Financial Systems. Journal of Contemporary Financial Research. 2022;5(01):63–76.

[61] Wu Q, Tang M, Xiao D, Wei B. Green Finance, Carbon Emission Reduction and Financial Risk——From the Perspective of Carbon Emission Reduction Scenario and Risk Mitigation. Review of Economic Research. 2023;(08):45–62. doi: 10.16110/j.cnki.issn2095-3151.2023.08.011

[62] Zhao X, Wang R, Lu D, Xue J, Cui X. The Macroeconomic Effects of Green Fiscal and Financial Policies under the ‘30·60’ Target: Based on a Stock-Flow Consistent Model. Shanghai Finance. 2022;(06):12–22. doi: 10.13910/j.cnki.shjr.2022.06.002

[63] Xie L, Zhou F, Chen S, Sun Q, Wang K. Analysis of the Impact of Climate Change on Financial Stability Based on the SVAR Model——Taking the Chinese Banking Industry as an Example. Fujian Finance. 2022;(12):3–13.

[64] Chen Y, Ren Y-S, Narayan S, Huynh NQA. Does climate risk impact firms’ ESG performance? Evidence from China. Economic Analysis and Policy. 2024;81:683–95.

[65] WANG W. Research Progress and Prospects of Financial Risks Under Climate Impacts: A Perspective Based on Physical Risks and Transition Risks. Finance & Economy. 2024;(04):34–51.

[66] WANG Z, NIU Y, REN X. How Climate Change Affects Systemic Financial Risk: Evidence from Extreme Climate Events and Green (Brown) Assets. China Journal of Econometrics. 2024;4(04):1009–30.

[67] Zhang Y, Guo K, Ji Q, Zhao W. Information Spillover Effect of Climate Shocks on Chinese Asset Returns. China Journal of Econometrics. 2023;3(02):426–42.

[68] Arouri MEH, Lahiani A, Lévy A, Nguyen DK. Forecasting the conditional volatility of oil spot and futures prices with structural breaks and long memory models. Energy Economics. 2012;34(1):283–93.

[69] Kang SH, Yoon S-M. Modeling and forecasting the volatility of petroleum futures prices. Energy Economics. 2013;36:354–62.

[70] Narayan PK, Narayan S. Modelling oil price volatility. Energy policy. 2007;35(12):6549–53.

[71] Sadorsky P. Modeling and forecasting petroleum futures volatility. Energy economics. 2006;28(4):467–88.

[72] Sévi B. Forecasting the volatility of crude oil futures using intraday data. European Journal of Operational Research. 2014;235(3):643–59.

[73] Andersen TG, Bollerslev T. Towards a unified framework for high and low frequency return volatility modeling. Statistica Neerlandica. 1998;52(3):273–302.

[74] Corsi F. A simple approximate long-memory model of realized volatility. Journal of Financial Econometrics. 2009;7(2):174–96.

[75] Andersen TG, Bollerslev T, Diebold FX. Roughing it up: Including jump components in the measurement, modeling, and forecasting of return volatility. The review of economics and statistics. 2007;89(4):701–20.

[76] Corsi F, Pirino D, Reno R. Threshold bipower variation and the impact of jumps on volatility forecasting. Journal of Econometrics. 2010;159(2):276–88.

[77] Zhou W-X, Sornette D. Non-parametric determination of real-time lag structure between two time series: The “optimal thermal causal path” method with applications to economic data. Journal of Macroeconomics. 2006;28(1):195–224.

[78] Gong C-C, Ji S-D, Su L-L, Li S-P, Ren F. The lead–lag relationship between stock index and stock index futures: A thermal optimal path method. Physica A: Statistical Mechanics and its Applications. 2016;444:63–72.

[79] Wang D, Tu J, Chang X, Li S. The lead–lag relationship between the spot and futures markets in China. Quantitative Finance. 2017;17(9):1447–56.

[80] Xu H-C, Zhou W-X, Sornette D. Time-dependent lead-lag relationship between the onshore and offshore Renminbi exchange rates. Journal of International Financial Markets, Institutions and Money. 2017;49:173–83.

[81] Yuan X, Jin L, Lian F. The lead–lag relationship between Chinese mainland and Hong Kong stock markets. Physica A: Statistical Mechanics and its Applications. 2021;574:125999.

[82] Andersen TG, Bollerslev T. Answering the skeptics: Yes, standard volatility models do provide accurate forecasts. International economic review. 1998:885–905.

[83] Andersen TG, Bollerslev T, Huang X. A reduced form framework for modeling volatility of speculative prices based on realized variation measures. Journal of Econometrics. 2011;160(1):176–89.

[84] Gong X, Lin B. Structural breaks and volatility forecasting in the copper futures market. Journal of Futures Markets. 2018;38(3):290–339.

[85] Zhang H, Zhu X, Guo Y, Liu H. A separate reduced‐form volatility forecasting model for nonferrous metal market: Evidence from copper and aluminum. Journal of Forecasting. 2018;37(7):754–66.

[86] Li X, Shen D, Zhang W. Do Chinese internet stock message boards convey firm-specific information? Pacific-Basin Finance Journal. 2018;49:1–14.

[87] Liang C, Tang L, Li Y, Wei Y. Which sentiment index is more informative to forecast stock market volatility? Evidence from China. International Review of Financial Analysis. 2020;71:101552.

[88] Wang G-J, Xiong L, Zhu Y, Xie C, Foglia M. Multilayer network analysis of investor sentiment and stock returns. Research in International Business and Finance. 2022;62:101707.

[89] Zhang Z, Su Z, Wang K, Zhang Y. Corporate environmental information disclosure and stock price crash risk: Evidence from Chinese listed heavily polluting companies. Energy Economics. 2022;112:106116.

[90] Asriadie MS, Mubarok MS, editors. Classifying emotion in Twitter using Bayesian network. Journal of Physics: Conference Series; 2018: IOP Publishing.

[91] Huang P-C, Wu J-S, Lee C-N, editors. Negative emotion event detection for Chinese posts on Facebook. 2015 International Conference on Cloud Computing and Big Data (CCBD); 2015: IEEE.

[92] Ren F, Kang X. Employing hierarchical Bayesian networks in simple and complex emotion topic analysis. Computer Speech & Language. 2013;27(4):943–68.

[93] Santi C. Investor climate sentiment and financial markets. International Review of Financial Analysis. 2023;86:102490.

[94] Hachicha F, Argoubi M, Guesmi K. The knowledge domain and emerging trends in Behavioral Finance: A Scientometric Analysis. Research in International Business and Finance. 2024:102404.

[95] Xu Y, Duong D, Xu H. Attention! Predicting crude oil prices from the perspective of extreme weather. Finance Research Letters. 2023;57:104190.

[96] Sui B, Chang C-P, Jang C-L, Gong Q. Analyzing causality between epidemics and oil prices: Role of the stock market. Economic Analysis and Policy. 2021;70:148–58.

[97] Ramos GM, Daamen W, Hoogendoorn S. A state-of-the-art review: Developments in utility theory, prospect theory and regret theory to investigate travellers’ behaviour in situations involving travel time uncertainty. Transport Reviews. 2014;34(1):46–67.

[98] Spencer N. Prospect theory and the potential for lottery-based subsidies. Behavioural Public Policy. 2023;7(2):500–17.

[99] Lee W, Choi YH, Kim C, Ahn JY. A case study for intercontinental comparison of herd behavior in global stock markets. Communications for Statistical Applications and Methods. 2018;25(2):185–97.

[100] Del Guayo Castiella I, Marmolejo Cervantes MA. The recovery of the energy sector after the COVID-19 pandemic: a comparison between Latin America and the European Union. Journal of Energy & Natural Resources Law. 2022;40(2):165–81.

[101] Hoffmann L, Bressem KK, Cittadino J, Rueger C, Suwalski P, Meinel J, et al. From Global Health to Global Warming: Tracing Climate Change Interest during the First Two Years of COVID-19 Using Google Trends Data from the United States. Environments. 2023;10(12):221.

[102] Ullah S, Khattak SR, Ullah R, Fayaz M, Han H, Yoo S, et al. Unveiling the global nexus: Pandemic fear, government responses, and climate change-an empirical study. Heliyon. 2024;10(1). doi: 10.1016/j.heliyon.2023.e23815 38261913 PMC10797138

[103] Umamah M, Mufti A, Ali K, Jilani S, Farooqi I, Khan M, et al. Navigating the Nexus: Insights from the COVID-19 Pandemic for Climate Change Mitigation. Asian Journal of Water, Environment and Pollution. 2024;21(3):27–34.

[104] Li X. Dynamic spillovers between US climate policy uncertainty and global foreign exchange markets: the pass-through effect of crude oil prices. Letters in Spatial and Resource Sciences. 2022;15(3):665–73. 36118955 10.1007/s12076-022-00318-4 PMC9466306

[105] Ding Y, Liu Y, Failler P. The impact of uncertainties on crude oil prices: based on a quantile-on-quantile method. Energies. 2022;15(10):3510.

[106] Chen Y, Zhang L, Zhang F. Forecasting crude oil volatility and stock volatility: New evidence from the quantile autoregressive model. The North American Journal of Economics and Finance. 2024;74:102235.

[107] Haukvik N, Cheraghali H, Molnár P. The role of investors’ fear in crude oil volatility forecasting. Research in International Business and Finance. 2024;70:102353.

[108] Herrera AM, Hu L, Pastor D. Forecasting crude oil price volatility. International Journal of Forecasting. 2018;34(4):622–35.

[109] Song Y, He M, Wang Y, Zhang Y. Forecasting crude oil market volatility: A newspaper-based predictor regarding petroleum market volatility. Resources Policy. 2022;79:103093.

[110] Zhang YJ, Zhang JL. Volatility forecasting of crude oil market: A new hybrid method. Journal of Forecasting. 2018;37(8):781–9.

[111] Liu W, Zhao P, Luo Z, Tang M. The dynamic impact of network attention on natural resources prices in pre-and post-Russian-Ukrainian war. Resources Policy. 2024;97:105271.

